# EDN1 and NTF3 in keloid pathogenesis: computational and experimental evidence as novel diagnostic biomarkers for fibrosis and inflammation

**DOI:** 10.3389/fgene.2025.1516451

**Published:** 2025-02-20

**Authors:** Hui Gong, Jing Liu, Nanji Chen, Hengguang Zhao, Bailin He, Hongpei Zhang, Wenping Wang, Yi Tian

**Affiliations:** ^1^ Department of Dermatology and Medical Aesthetics, The Second Affiliated Hospital of Chongqing Medical University, Chongqing, China; ^2^ Department of Pathogen Biology, College of Basic Medical Science, Chongqing Medical University, Chongqing, China; ^3^ Center of Medical Cosmetology, The People’s Hospital of Wusheng, Chongqing, China

**Keywords:** keloid formation, oxidative stress, fibrosis, inflammation, immune response, MAPK, EDN1, NTF3

## Abstract

**Objective:**

To investigate the roles of oxidative stress-related differentially expressed genes (OSRDEGs) in keloid formation and explore their potential value in diagnosis and treatment.

**Methods:**

Gene expression data from the GEO database, including GSE145725 and GSE44270 as training sets and GSE7890 as a validation set, were utilized. OSRDEGs were identified, followed by Weighted Gene Co-expression Network Analysis (WGCNA), GO/KEGG enrichment analysis, and Gene Set Enrichment Analysis (GSEA). Key genes were further screened through protein-protein interaction (PPI) network analysis and receiver operating characteristic (ROC) curve analysis. miRNA targets, transcription factors (TF), and potential drug targets of these genes were predicted. Immune cell infiltration analysis was performed to assess the association between OSRDEGs and immune cells, which was validated using GSE7890. Finally, the expression of key genes was experimentally validated using quantitative PCR (qPCR), immunohistochemistry (IHC), and hematoxylin-eosin (HE) staining.

**Results:**

A total of 13 OSRDEGs were identified. WGCNA and functional enrichment analyses revealed that these genes were primarily involved in fibrosis and inflammatory processes in keloids, such as the MAPK signaling pathway, lymphocyte and monocyte proliferation, and inflammatory pathways involving IL-18 and IL-23. PPI network analysis, ROC analysis, and immune infiltration results identified Endothelin-1 (EDN1) and Neurotrophin-3(NTF3) as key genes with high sensitivity and specificity. These genes were positively and negatively correlated with activated mast cells, respectively, suggesting their dual regulatory roles in fibrosis and inflammation. External dataset validation, qPCR, correlation analysis, HE staining, and IHC results demonstrated that EDN1 and NTF3 were highly expressed in keloid tissues and were associated with excessive collagen deposition and immune cell infiltration.

**Conclusion:**

EDN1 and NTF3, as OSRDEGs, play critical roles in the pathogenesis and progression of keloids. They may contribute to fibrosis and inflammation through the regulation of oxidative stress, the MAPK signaling pathway, and mast cell activation. These findings highlight EDN1 and NTF3 as potential diagnostic biomarkers and therapeutic targets, providing novel insights into the pathogenesis and treatment strategies for keloids.

## 1 Introduction

Keloid is a typical fibroproliferative skin disorder characterized by excessive scar tissue formation that extends beyond the original wound boundary, causing persistent itching, pain, and cosmetic disfigurement, which severely impacts patients’ quality of life (Ogawa, 2017). Pathologically, keloids are often associated with a dual pathology of chronic inflammation and excessive fibrosis (Shih et al., 2010). On the one hand, fibroblasts are persistently activated, synthesizing and depositing extracellular matrix (ECM) components such as collagen, resulting in significant thickening of the dermis (Darby et al., 2014). On the other hand, immune cells such as macrophages, mast cells, and T lymphocytes excessively infiltrate the keloid tissue, releasing inflammatory cytokines that exacerbate disease progression (Shih et al., 2010). However, there is ongoing debate regarding whether fibrosis and inflammation are primary pathological drivers of keloid formation or secondary effects of upstream genetic or signaling disruptions (Jiang and Rinkevich, 2020). Given the significant ethnic differences and genetic predispositions in keloid formation, as well as the lack of ideal animal models, elucidating its precise pathogenic mechanisms remains challenging (Nakashima et al., 2010).

In recent years, OSRDEGs have been increasingly recognized for their roles in various fibrosis-related diseases and skin pathologies (Antar et al., 2023). For example, systemic sclerosis (SSc) is a systemic fibrotic disease characterized by extensive fibrosis of the skin and internal organs (Allanore et al., 2015). Studies have shown that overexpression of OSRDEG NOX4, which encodes NADPH oxidase responsible for reactive oxygen species (ROS) production, plays a critical role in the pathogenesis of SSc (Hecker and Thannickal, 2011). Excessive ROS disrupts intracellular antioxidant balance, causing direct damage to lipids, proteins, and DNA, ultimately leading to cellular dysfunction (Forman et al., 2009). Furthermore, ROS can activate the TGF-β signaling pathway and promote fibroblast proliferation and collagen deposition through SMAD3 regulation (Miyazono, 2000). Therefore, OSRDEGs exacerbate fibrosis in SSc by both directly damaging cells via excessive ROS production and activating fibrotic signaling pathways, leading to widespread tissue fibrosis (Hecker et al., 2014). In idiopathic pulmonary fibrosis (IPF), Armanios et al. demonstrated that mutations in TERC and TERT genes lead to telomere shortening, triggering cellular senescence (Armanios and Blackburn, 2012). Senescent cells with impaired antioxidant defenses are more susceptible to oxidative stress-induced damage (Kuilman et al., 2010). The combined effects of telomere shortening and oxidative stress drive chronic inflammation and fibrosis, resulting in excessive fibroblast proliferation, abnormal ECM accumulation, and irreversible pulmonary fibrosis (Alder et al., 2011). Additionally, excessive oxidative stress disrupts various stages of wound healing, leading to chronic wounds (Schäfer and Werner, 2008). Oxidative stress elevates intracellular ROS levels, impairing mitochondrial function and energy metabolism, and directly inhibits fibroblast proliferation and migration (Kim and Choi, 2010). ROS can also oxidize DNA and proteins, causing cell cycle arrest at the G1/S phase, thereby delaying wound repair (Sundaresan et al., 2012). Moreover, ROS further enhances inflammatory responses by activating inflammation-related pathways, such as NF-κB, ultimately impeding wound healing (Morgan and Liu, 2011).

OSRDEGs have been shown to be closely associated with fibrosis and inflammation in these diseases, particularly through their ability to regulate immune cell function and ECM deposition (Hecker et al., 2014). Systemic fibrotic diseases and keloids share notable pathological similarities, both characterized by persistent fibrosis and chronic inflammation (Hinz, 2007). Therefore, we hypothesize that OSRDEGs may also contribute to keloid formation through similar mechanisms, potentially driven by oxidative stress. However, the specific roles and molecular mechanisms of OSRDEGs in keloid pathology remain poorly understood. This study aims to uncover the critical roles of OSRDEGs in keloid pathogenesis through a combination of bioinformatics analysis and experimental validation. The specific objectives include: (1) identifying OSRDEGs that are significantly differentially expressed in keloid tissues; (2) investigating the key signaling pathways and molecular mechanisms by which these genes regulate fibrosis and inflammatory responses in keloids; and (3) evaluating the clinical utility of key OSRDEGs as potential diagnostic biomarkers and therapeutic targets for keloids. By integrating bioinformatics analysis with experimental validation, this innovative research strategy avoids the limitations of relying solely on a single approach, providing novel insights into the regulatory mechanisms and clinical potential of OSRDEGs.

## 2 Materials and methods

### 2.1 Acquisition of data

Gene expression profiles from keloid patients were obtained from the Gene Expression Omnibus (GEO) database (http://www.ncbi.nlm.nih.gov/geo) using datasets GSE145725 and GSE44270, both derived from *Homo sapiens*. GSE145725 includes microarray data from skin and scar tissues, comprising 19 samples: 9 keloid fibroblast samples (Keloid group) and 10 normal fibroblast samples (Normal group). The analysis was performed using the GPL16043 GeneChip^®^ PrimeView™ Human Gene Expression Array platform. GSE44270 consists of microarray gene expression profiles from skin and scar tissues, including a total of 12 samples: 9 keloid fibroblasts (Keloid group) and 3 normal fibroblasts (Normal group). The data were generated using the GPL6244 (HuGene-1_0-st) Affymetrix Human Gene 1.0 ST Array platform.

### 2.2 Data set merging and correction

To integrate the GSE145725 and GSE44270 datasets, we first identified common genes present in both datasets. After aligning the datasets based on these common genes, they were merged into a single expression matrix. The batch effects between the two datasets were corrected using the ‘ComBat’ function from the ‘sva’ package in R (version 3.48.0). This step was crucial to ensure consistency in the Combined-dataset, enabling accurate downstream analyses.

Boxplots were generated to visualize the distribution of expression values across samples before and after batch correction, demonstrating that the batch effects were effectively eliminated in the merged dataset.

### 2.3 Identification and visualization OSRDEGs in keloid and normal samples

After batch correction, differential expression analysis was conducted on the combined dataset of 18 keloid and 13 normal samples using the ‘limma’ package in R (version 3.56.2). A linear model was used to compare the keloid and normal groups, with contrast matrices generated to evaluate expression differences. Differentially expressed genes (DEGs) were identified based on the criteria: |logFC| > 1 and adjusted P-value <0.05. Genes with logFC >1 were classified as upregulated, while those with logFC < −1 were classified as downregulated.

A predefined list of 1,596 oxidative stress-related genes (OSRGs) was obtained from the GeneCards website (https://www.genecards.org/, Version 5.21, updated on August 5, 2024). The DEGs were then intersected with these OSRGs to identify OSRDEGs. The OSRDEGs were then visualized using Venn diagrams, volcano plots, differential sorting plots, heatmaps, and a chromosome location map, all of which were generated using the Xiantaoxueshu online tool (www.xiantao.love, version 2022.1).

#### 2.3.1 Functional enrichment analyses for OSRDEGs

Gene Ontology (GO) analysis, including categories such as Biological Process (BP), Molecular Function (MF), and Cellular Component (CC), along with Kyoto Encyclopedia of Genes and Genomes (KEGG) pathway analysis, as well as the integrated GO-KEGG analysis incorporating LogFC values, were performed to investigate the functional enrichment and pathways associated with the identified OSRDEGs. These analyses were conducted using the Xiantaoxueshu online tool. The criteria for statistical significance were set at *p* < 0.05, with false discovery rate (FDR) values (q.value) < 0.25, as adjusted by the Benjamini–Hochberg (BH) method.

#### 2.3.2 Gene set enrichment analysis (GSEA)

GSEA was employed to assess the distribution of predefined gene sets across a ranked list of genes, ordered by their relevance to the keloid phenotype. The GSEA was performed on the Combined-Dataset, which was divided into Keloid and Normal groups. The analysis was conducted using the Xiantaoxueshu online tool, applying a reference gene set: c2. cp.all.v2022.1. Hs.symbols.gmt (All Canonical Pathways). For this analysis, the parameters included 1,000 permutations and a focus on gene sets with a minimum of 10 and a maximum of 500 genes. Selection criteria for significant gene sets were *p* < 0.05 and FDR <0.25.

#### 2.3.3 Weighted gene Co-Expression network analysis (WGCNA)

WGCNA is a systems biology approach used to describe the correlation patterns among genes across multiple samples. It enables the identification of gene modules, which are clusters of highly correlated genes, and relates these modules to external traits or phenotypes, such as fibrosis and inflammation in this study. The analysis begins with constructing a gene co-expression network by calculating pairwise correlations between gene expression levels and applying a soft-thresholding power to ensure scale-free topology. Gene modules are then identified using hierarchical clustering, followed by eigengene-based module summarization. Finally, module-trait relationships are assessed to identify modules significantly associated with the clinical or biological traits of interest. In this study, WGCNA was used to identify modules strongly correlated with fibrosis and inflammation, providing insights into the regulatory roles of key genes in keloid pathogenesis.

### 2.4 PPI network analysis

The STRING database (https://string-db.org/, version 11.0) was utilized to construct a PPI network, identifying interactions among the OSRDEGs. Key hub genes were identified based on their connectivity within the network. Furthermore, the GeneMANIA tool (http://genemania.org/, version 3.6) was employed to predict networks of genes functionally related to these hub genes, providing additional insights into their interactions.

### 2.5 ROC analysis of hub genes

ROC analysis was carried out to evaluate the diagnostic accuracy of these hub genes. The Area Under the Curve (AUC) values were calculated to determine the potential of these genes in distinguishing keloid from normal tissues, further refining the selection of hub genes with high diagnostic relevance.

### 2.6 Prediction of miRNA, transcription Factors (TFs), and drug-gene interaction networks

To investigate the interactions between hub genes and miRNAs, we used the miRDB database (http://mirdb.org, version 6.0). For TFs interacting with hub genes, the CHIPBase database (https://rna.sysu.wsu.cn/chipbase/, version 3.0) and the HTFtarget database (http://bioinfo.life.hust.edu.cn/hTFtarget/) were consulted. Additionally, drug-gene interaction analysis was performed using the Comparative Toxicogenomics Database (CTD, http://ctdbase.org, version 5.0) to identify potential therapeutic targets. All interaction networks, including mRNA-TF and drug-gene interactions, were visualized using Cytoscape software (version 3.10.1).

### 2.7 qPCR analysis

Quantitative polymerase chain reaction (qPCR) was conducted to measure the mRNA expression levels of genes in keloid and normal skin tissues. Total RNA was extracted using TRizol reagent (Invitrogen). cDNA was synthesized using the PrimeScript™ RT Reagent Kit with gDNA Eraser (Takara) according to the manufacturer’s protocol. qPCR was performed using the 2× Universal SYBR Green Fast qPCR Mix (Abclonal) with the following reaction conditions: 1 cycle of 95°C for 30 min, 40 cycles of 95°C for 5 s, and 60°C for 30 s. All PCR primers were designed using NCBI Primer (Supplementary Table S1) and were synthesized by Beijing Tsingke Biotech Co., Ltd. The relative expression levels of various genes were analyzed using qPCR, comparing the expression between keloid and normal skin tissues.

#### 2.7.1 Histological and clinical analysis

A comparative clinical and histological analysis was performed to identify structural and cellular differences between keloid tissue and normal skin. Clinical photographs documented the macroscopic differences between the two tissue types. Subsequently, tissue sections were stained using hematoxylin and eosin (H&E) to examine microscopic structures, focusing on collagen fiber organization and epidermal cell arrangement. Sections were imaged with KFBIO Digital Pathology Slide Scanner and were observed under 10x and 40x magnifications, revealing marked differences in tissue architecture between keloid and normal skin, including disorganized collagen fibers and irregular cell structures characteristic of keloid tissue.

#### 2.7.2 IHC analysis

For immunohistochemical staining of keloid and normal skin, tissues sections were permeabilized in 0.3% Triton X-100 for 10 min, and blocked with 5% BSA in PBS for 1 h at RT. Tissues were then incubated on a shaker with the appropriate primary antibody: EDN1 antibody (Proteintech, 12191-1-AP, 1:100) and NTF3 (Proteintech, 18084-1-AP, 1:100). Sections were imaged with KFBIO Digital Pathology Slide Scanner and were analyzed at 10x and 40x magnifications to assess the localization and intensity of protein expression. Differences in staining patterns between keloid and normal skin tissues were used to infer the possible roles of these proteins in the development and progression of keloids.

### 2.8 Statistical analysis

**FIGURE 1:**
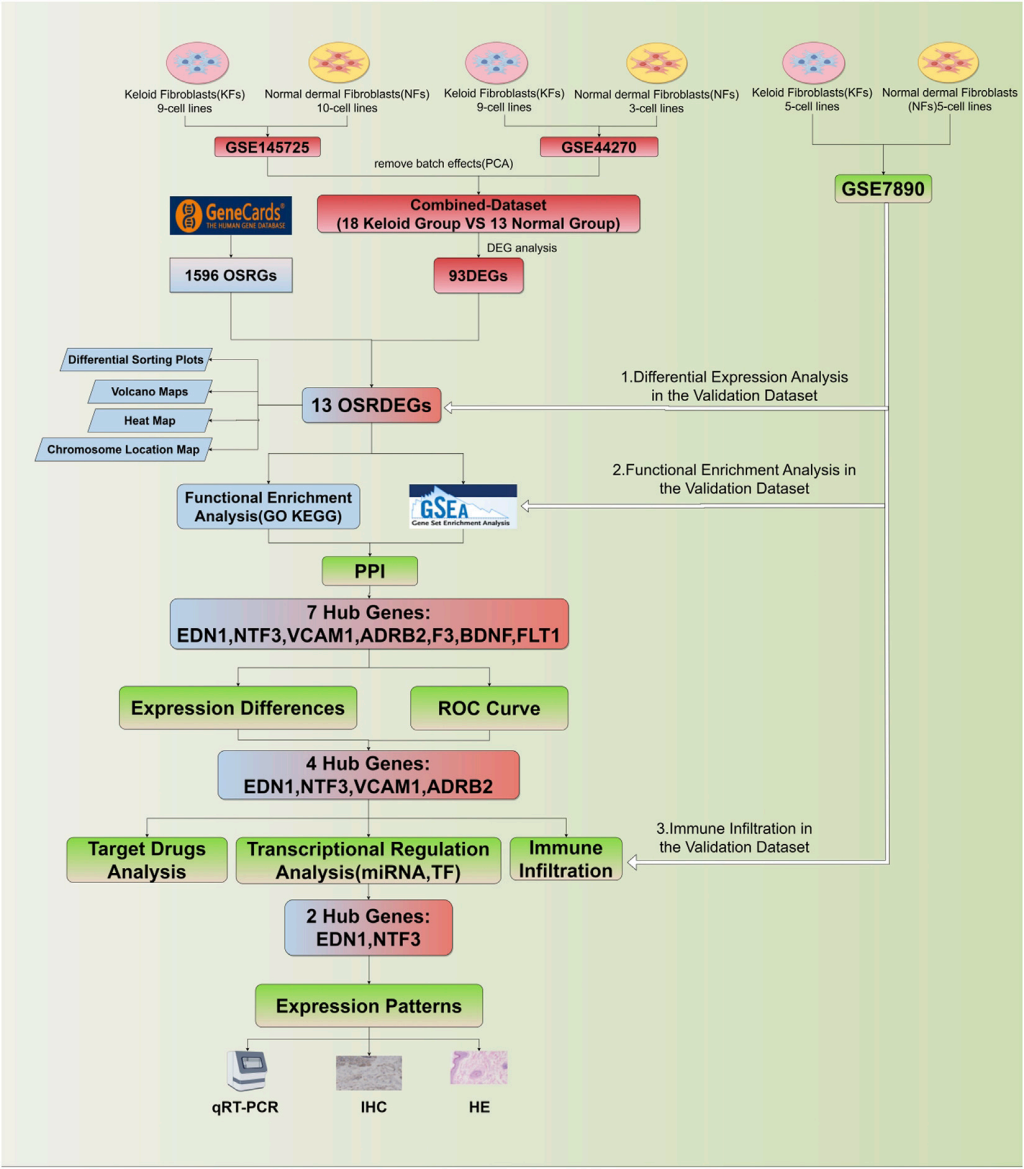
Flowchart of this study.

All statistical analyses were conducted using R software (version 4.2.1) to assess gene expression differences and their diagnostic relevance. Gene expression differences between groups were evaluated using t-tests for two-group comparisons, while one-way ANOVA was applied for comparisons across multiple groups. Correlations between gene expression and immune cell infiltration were assessed using Pearson or Spearman correlation coefficients, depending on the data distribution. A *p* < 0.05 was considered statistically significant, and 95% confidence intervals (CI) were provided for key metrics to quantify the uncertainty of estimates. The research flowchart for this study is shown in Figure 1.

## 3 Results

### 3.1 Batch correction and identification of OSRGs

Boxplots were generated to visualize the distribution of expression values across samples before and after batch correction, demonstrating that the batch effects were effectively eliminated in the merged dataset (Supplementary Figure S1). Additionally, a predefined list of 1,596 oxidative stress-related genes (OSRGs) was obtained from the GeneCards website (Supplementary Table S1).

### 3.2 Expression patterns of key OSRDEGs

**FIGURE 2:**
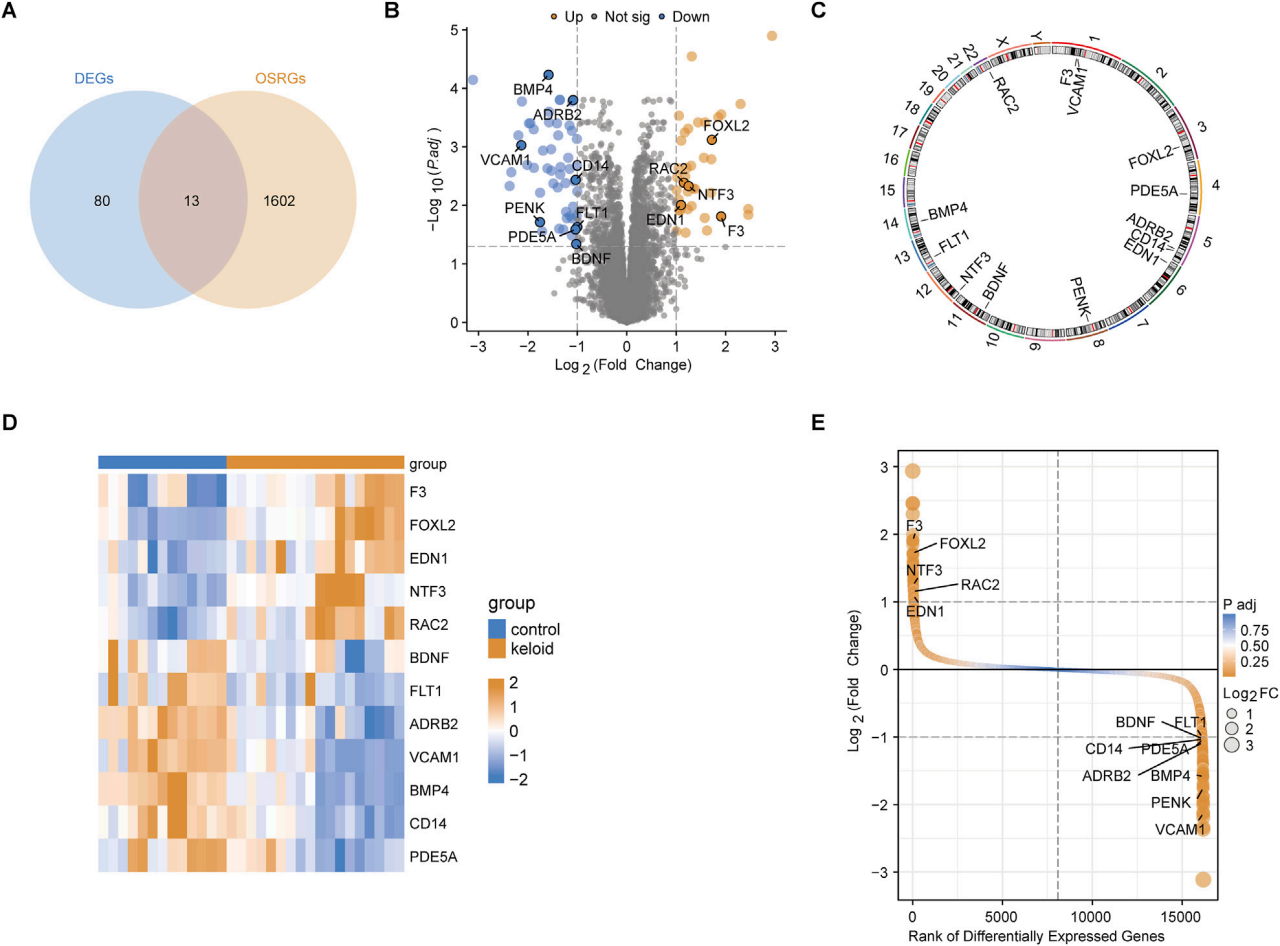
Identification and characterization of OSRDEGs. **(A)** The Venn diagram identifies 13 OSRDEGs through intersection analysis; **(B)** The volcano plot illustrates the differential expression of DEGs between the keloid and normal skin groups, with significant genes (such as *F3, FOXL2,* and *EDN1*) showing a *Log2 Fold Change > 2* and *P-value < 0.01*; **(C)** Chromosomal location map; **(D)** The heatmap displays the expression differences of the 13 OSRDEGs between keloid and normal skin tissues; **(E)** The ranked plot orders DEGs based on *Log2 Fold Change* and statistical significance, highlighting OSRDEGs with notable differential expression, with *F3, FOXL2,* and *NTF3* showing prominent rankings and large expression changes.

Through the intersection analysis of DEGs and OSRGs, 13 OSRDEGs were identified (Figure 2A). These genes are prominently highlighted in the volcano plot (Figure 2B) and are mainly located on chromosomes 1 and 5 (Figure 2C). The heatmap illustrates the differential expression of these 13 OSRDEGs between keloid and normal skin tissues (Figure 2D), where F3, FOXL2, EDN1, and NTF3 show higher expression in keloid tissues, while BDNF, FLT1, VCAM1 and ADRB2 are more highly expressed in normal skin. Additionally, the ranked plot of differential expression shows that F3, FOXL2, NTF3 and EDN1 exhibit larger fold changes and higher statistical significance, whereas BDNF and FLT1 show smaller expression changes (Figure 2E).

### 3.3 GO and KEGG analysis of OSRDEGs

**FIGURE 3:**
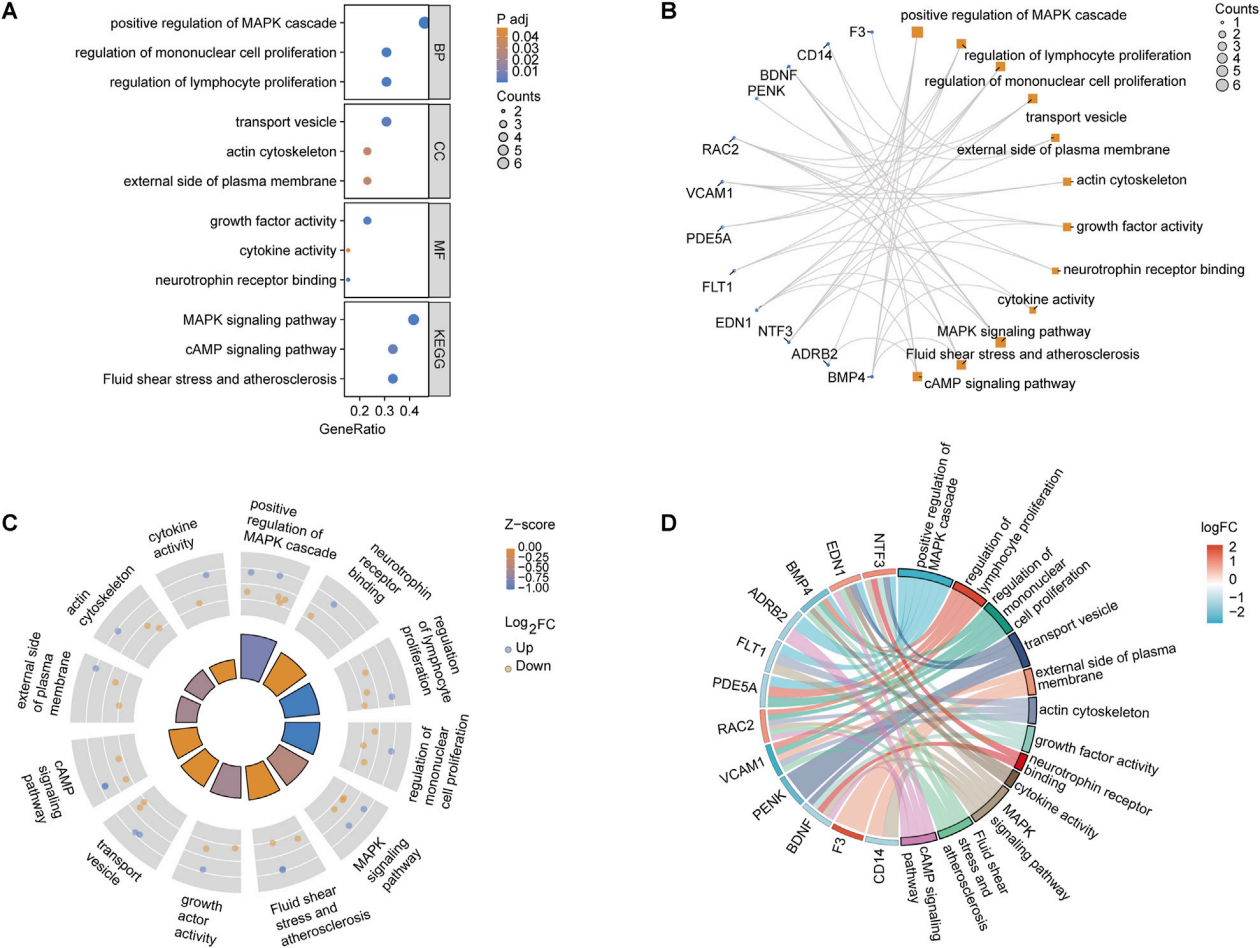
GO and KEGG pathway enrichment analysis of OSRDEGs. **(A)** Bar graph showing the *GeneRatio* and *p*-values, illustrating the most significantly enriched biological processes, cellular components, and molecular functions. **(B)** Network plot displaying the association between OSRDEGs and enriched GO terms and KEGG pathways, with larger nodes representing genes associated with multiple terms and pathways. **(C)** Circle plot integrating *logFC* and *Z-scores*, highlighting significantly enriched key pathways. **(D)** Chord diagram visualizing the relationship between OSRDEGs and major enriched pathways, with colors representing changes in *logFC*.

GeneRatio analysis revealed significant enrichment in biological processes, including the “positive regulation of the MAPK cascade” (*GeneRatio 0.4, p < 0.01*) and the “regulation of mononuclear cell proliferation” (*GeneRatio 0.35, p < 0.03*). Cellular components like the “external side of the plasma membrane” and molecular functions such as “neurotrophin receptor binding” and “growth factor activity” were also enriched. KEGG pathway analysis highlighted key pathways, such as “MAPK signaling” (*GeneRatio 0.3, p < 0.02*) and “fluid shear stress and atherosclerosis” (Figure 3A). The relationship between OSRDEGs and enriched GO and KEGG terms revealed that genes such as F3, FOXL2, NTF3, and EDN1 were particularly linked to these pathways, notably the “MAPK signaling pathway” (Figure 3B). The analysis combining logFC and Z-score underscored the enrichment of key biological processes, further emphasizing pathways such as “neurotrophin receptor binding” and “positive regulation of the MAPK cascade” (Figures 3C, D).

#### 3.3.1 GSEA

**FIGURE 4:**
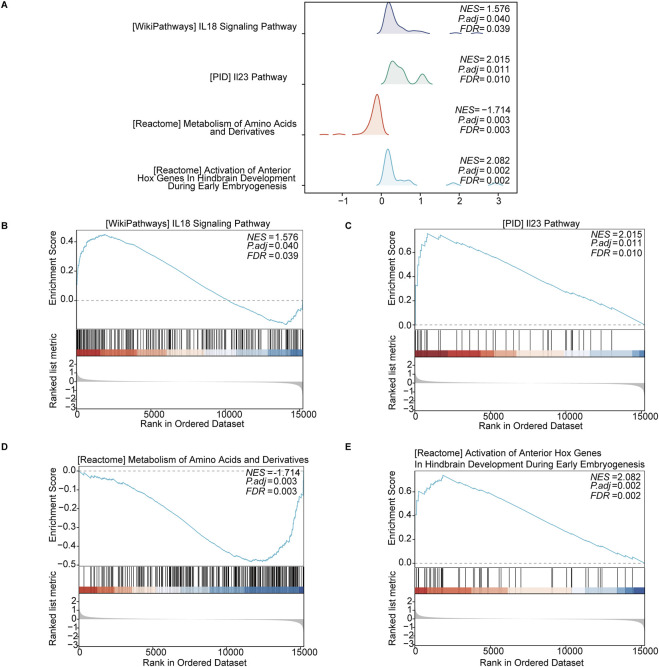
GSEA revealed significantly enriched pathways in keloid tissue. **(A)** Enrichment curves for four key pathways, including the “IL-18 signaling pathway,” “IL-23 signaling pathway,” “metabolism of amino acids and derivatives,” and “activation of anterior Hox genes in hindbrain development.” **(B–E)** Detailed enrichment plots for each pathway, showing the NES, *p*-adjusted values, FDR values, and the distribution of ranked genes in the ordered dataset.

The GSEA revealed significant enrichment in several key pathways in keloid tissue, including the “IL-18 signaling pathway” (NES = 1.576, *p* = 0.040, FDR = 0.039) and the “IL-23 signaling pathway” (NES = 2.015, *p* = 0.011, FDR = 0.010). Additionally, metabolic pathways such as the “metabolism of amino acids and derivatives” (NES = −1.714, *p* = 0.003, FDR = 0.003) were also significantly enriched. The “activation of anterior Hox genes in hindbrain development during early embryogenesis” (NES = −2.082, *p* = 0.002, FDR = 0.002) also showed significant enrichment. These enriched pathways indicate the involvement of OSRDEGs in various signaling and metabolic processes, with gene rank distribution showing significant gene placement within these pathways (Figures 4A–E).

#### 3.3.2 WGCNA

**FIGURE 5:**
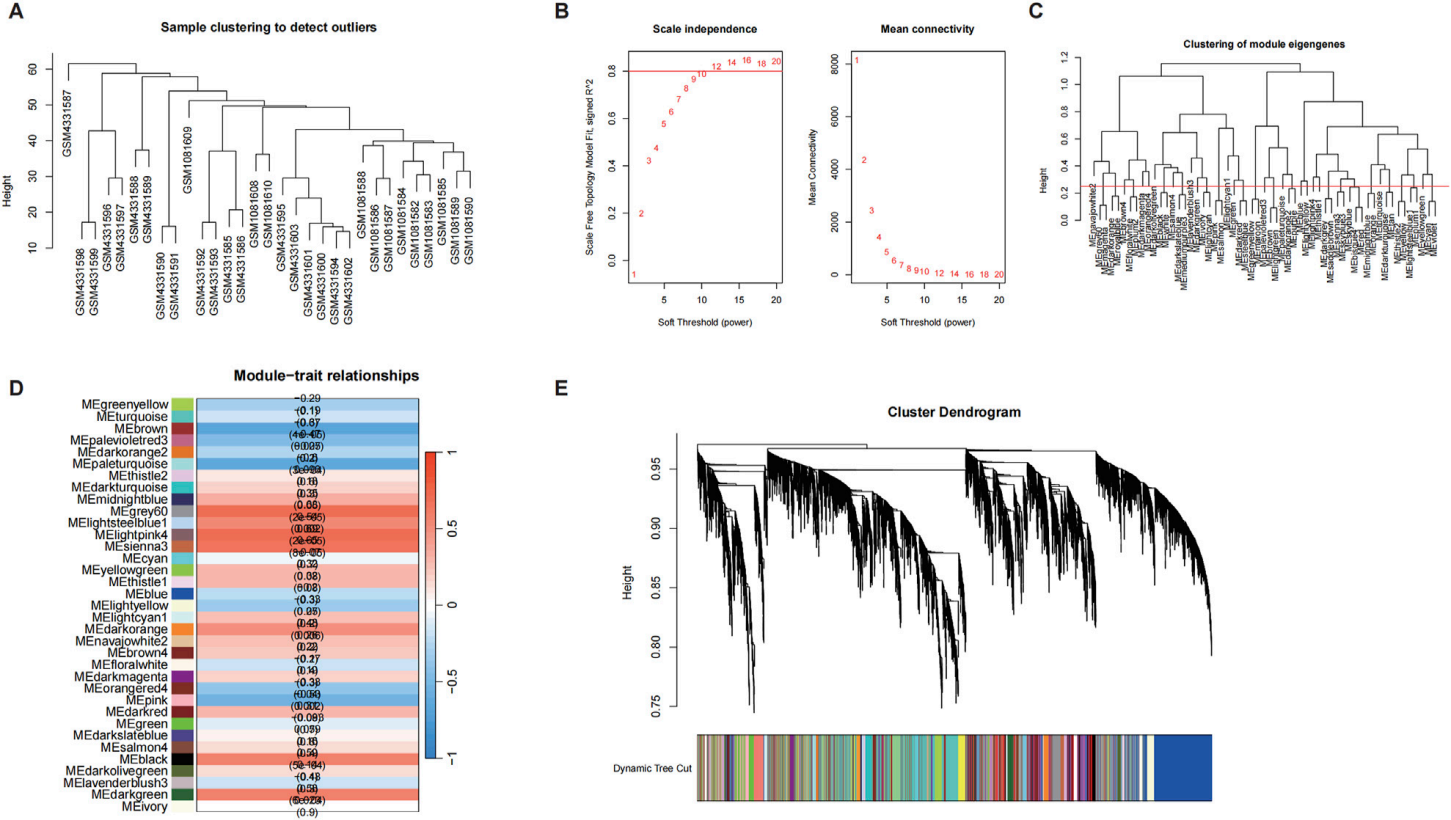
WGCNA Analysis Identifies Key Modules Associated with Fibrosis and Inflammation. WGCNA results identify modules associated with fibrosis and inflammation in keloid tissues. **(A)** Sample clustering to detect outliers. **(B)** Scale-free topology fit index (left) and mean connectivity (right) for different soft-thresholding powers used to construct the network. **(C)** Clustering of module eigengenes to identify relationships between modules. **(D)** Heatmap of module-trait relationships showing the correlation between identified modules and clinical traits. EDN1 and NTF3 are located in the brown and blue modules, which are significantly associated with fibrosis and inflammation. Correlation coefficients (R) and p-values are presented in parentheses. **(E)** Dendrogram of genes clustered into co-expression modules, with each module represented by a unique color.

**FIGURE 6:**
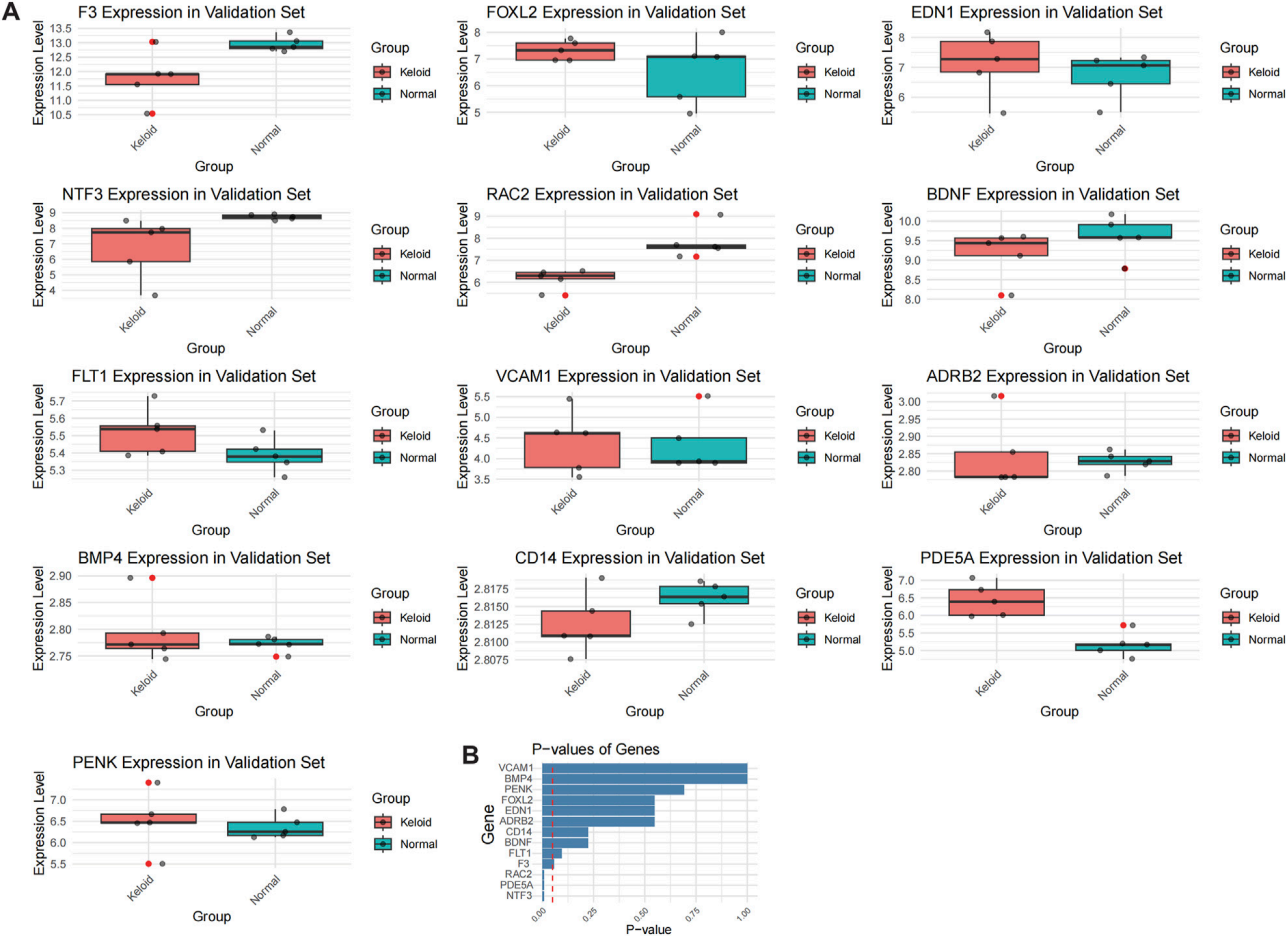
Validation of OSRDEG expression levels in GSE7890. **(A)** Box plots showing the expression levels of 13 OSRDEGs (F3, FOXL2, EDN1, NTF3, RAC2, BDNF, FLT1, VCAM1, ADRB2, BMP4, CD14, PENK, and PDE5A) in the keloid group (red) and normal tissue group (blue). Mann-Whitney U test results indicate that EDN1 was significantly upregulated in the validation dataset, while FLT1, FOXL2, and VCAM1 displayed consistent expression trends with the training datasets. Significant differences are marked with asterisks (*p < 0.05). **(B)** Bar plot showing the p-values of the genes in the validation dataset, ranked in ascending order of statistical significance.

**FIGURE 7:**
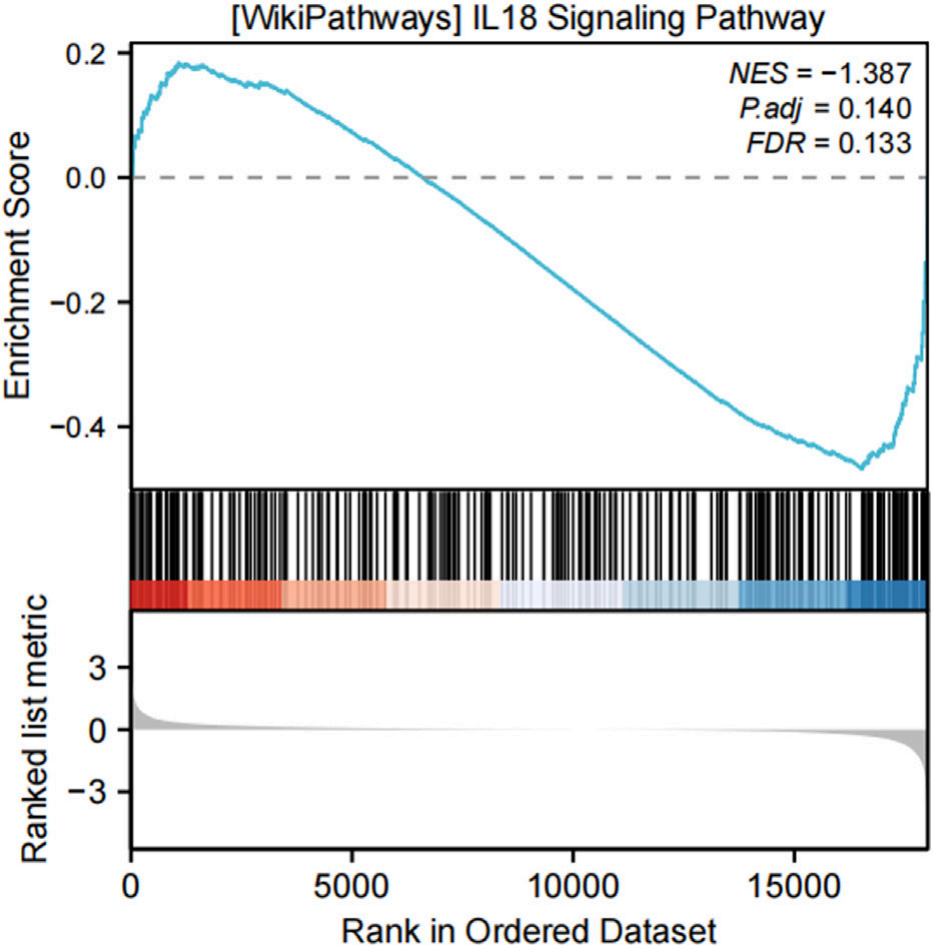
GSEA analysis of the IL-18 signaling pathway in the validation dataset GSE7890. The IL-18 signaling pathway was significantly enriched in both the training and validation datasets, reinforcing the reliability of the findings. The enrichment plot illustrates the distribution of IL-18 pathway genes in the ranked gene list, with the following metrics provided: normalized enrichment score (NES = −1.387), adjusted p-value (P.adj = 0.140), and false discovery rate (FDR = 0.133). These results highlight the critical involvement of the IL-18 signaling pathway in keloid formation.

**FIGURE 8:**
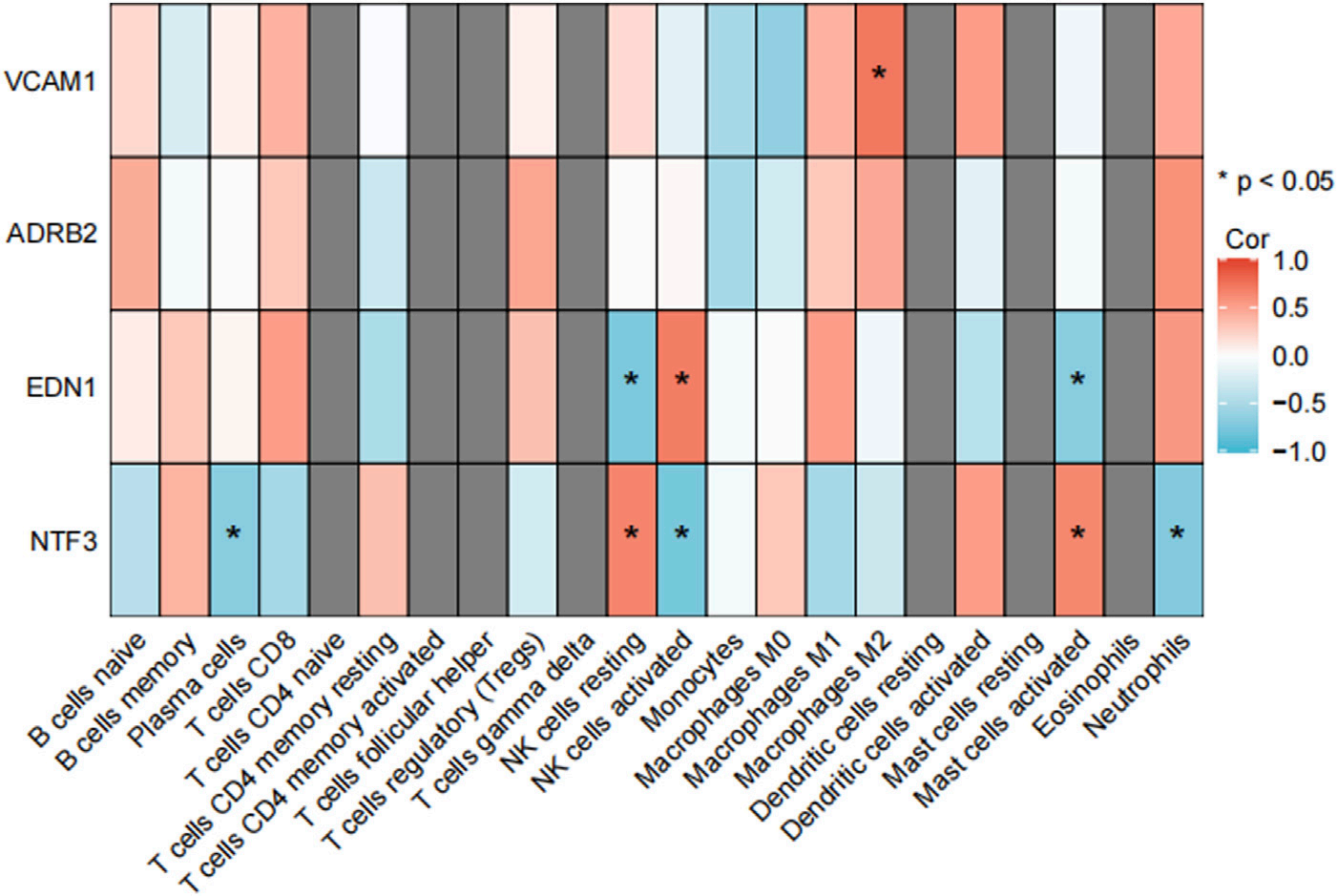
Correlation analysis between immune cells in GSE7890. NTF3 showed a significant positive correlation with activated mast cells in both the validation dataset GSE7890 and the training datasets. This finding further supports its dual regulatory role in fibrosis and inflammation. The heatmap illustrates the correlation coefficients between NTF3 and various immune cell types, with activated mast cells exhibiting the strongest positive correlation (*p < 0.05).

**FIGURE 9:**
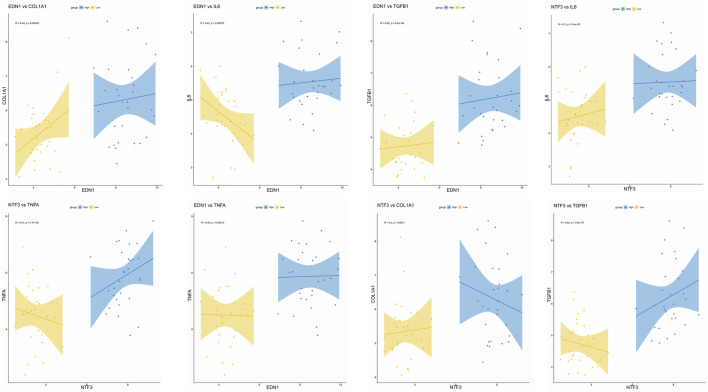
Correlation of EDN1 and NTF3 with Fibrosis Markers and Inflammatory Factors. Data support the significant positive correlations of EDN1 and NTF3 with fibrosis markers (COL1A1 and TGFB1) and inflammatory factors (IL6 and TNFA), indicating their pivotal roles in fibrosis and inflammation. Scatter plots show the relationships between EDN1/NTF3 expression levels and corresponding markers, with regression lines and 95% confidence intervals included. Correlation coefficients (R) and p-values are displayed for each relationship (*p < 0.05). High and low expression groups are represented in blue and yellow, respectively.

**FIGURE 10:**
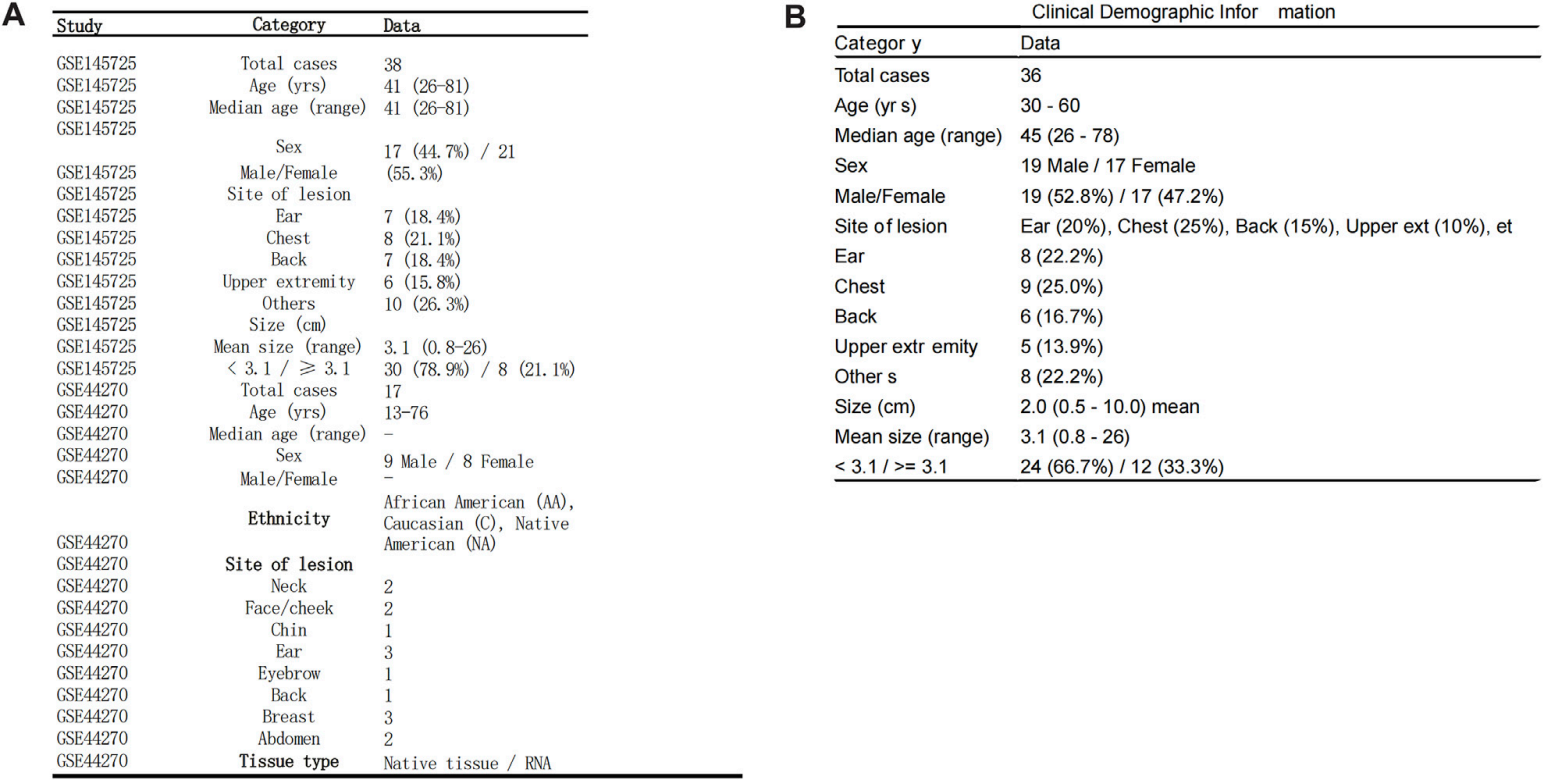
Clinical and Demographic Information of GEO Datasets. Clinical and demographic information of patients included in the GSE145725 and GSE44270 datasets, presented in tabular format. **(A)** Demographic details of the GSE145725 dataset, including age, sex distribution, lesion sites, and lesion sizes, are summarized. Ethnicity information is also included for the GSE44270 dataset. **(B)** Clinical data from our team’s self-collected keloid samples, including age, sex, lesion sites, and lesion sizes, further enhance the representativeness and transparency of the study. These datasets provide comprehensive demographic coverage, supporting the robustness of the study findings.

In the WGCNA analysis, we first performed sample clustering on the GSE145725 and GSE44270 datasets to identify and remove potential outliers, ensuring data quality. Subsequently, a soft-thresholding power of 6 was selected to construct a gene co-expression network that conformed to scale-free topology. Genes were grouped into multiple modules, among which the brown and blue modules showed significant correlations with fibrosis and inflammation phenotypes (R = 0.78, p < 0.01; R = 0.64, p < 0.05, respectively). Further analysis revealed that EDN1 and NTF3 were centrally located within these modules, serving as hub genes with high connectivity, indicating their pivotal roles in regulating fibrosis and inflammation. Additionally, module eigengenes were significantly positively correlated with external phenotypes, including fibrosis markers (COL1A1 and TGFB1) and inflammatory factors (IL6 and TNFA), further supporting the dual regulatory roles of EDN1 and NTF3 in these processes. These findings demonstrate that the WGCNA approach effectively identifies key genes associated with disease mechanisms, providing valuable insights into the molecular pathways underlying keloid pathogenesis. In addition to the WGCNA results (Figure 5) described above, we provided additional supporting data to further validate our findings (Figures 6–10). Specifically, Figure 6 shows that OSRDEGs are significantly differentially expressed between keloid and normal tissues in an independent dataset. Figure 7 demonstrates a significant enrichment of the IL-18 signaling pathway in keloid tissues. Figure 8 illustrates robust correlations between hub gene expression and immune cell infiltration. Figure 9 demonstrates that EDN1 and NTF3 are significantly positively correlated with fibrosis markers (COL1A1 and TGFB1) and inflammatory factors (IL6 and TNFA), underscoring their critical roles in fibrosis and inflammation. Finally, Figure 10 summarizes the clinical and demographic characteristics of the GEO datasets, underscoring the robustness of our data.

### 3.4 PPI network of OSRDEGs

**FIGURE 11:**
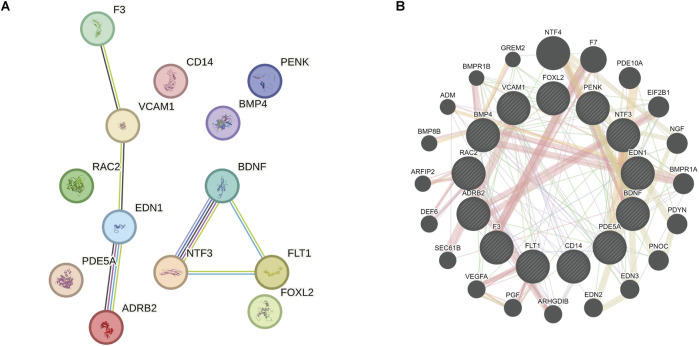
PPI network and GeneMANIA analysis revealing key genes and their interactions related to keloid formation. **(A)** PPI network analysis demonstrates the complex interactions among the 13 OSRDEGs and highlights central genes. The lines represent interactions between the genes. **(B)** GeneMANIA further refines these interactions, with line thickness indicating interaction strength. Seven hub genes were identified, which may play pivotal roles in the formation and progression of keloids.

PPI network analysis (minimum required interaction score = 0.700) revealed complex interactions among the 13 OSRDEGs, with EDN1 (interaction score = 0.85), ADRB2 (interaction score = 0.82), NTF3 (interaction score = 0.80), and BDNF (interaction score = 0.78) emerging as central nodes, suggesting their critical roles in regulating keloid-related pathways (Figure 11A). These interactions outline a complex signaling network essential for cellular communication and keloid pathology. GeneMANIA further refined this network (Figure 11B), where thicker lines represent stronger interactions between nodes. Key genes such as EDN1, ADRB2, NTF3, and BDNF once again emerged as central hubs, with significant interaction strengths. Ultimately, 7 hub genes—EDN1, NTF3, VCAM1, ADRB2, BDNF, F3 and FLT1—were identified, all of which had interaction scores above 0.70, indicating their potential roles in keloid formation and progression. These genes will be the focus of further investigation to determine their specific contributions to the disease.

### 3.5 ROC diagnosis of hub genes

**FIGURE 12:**
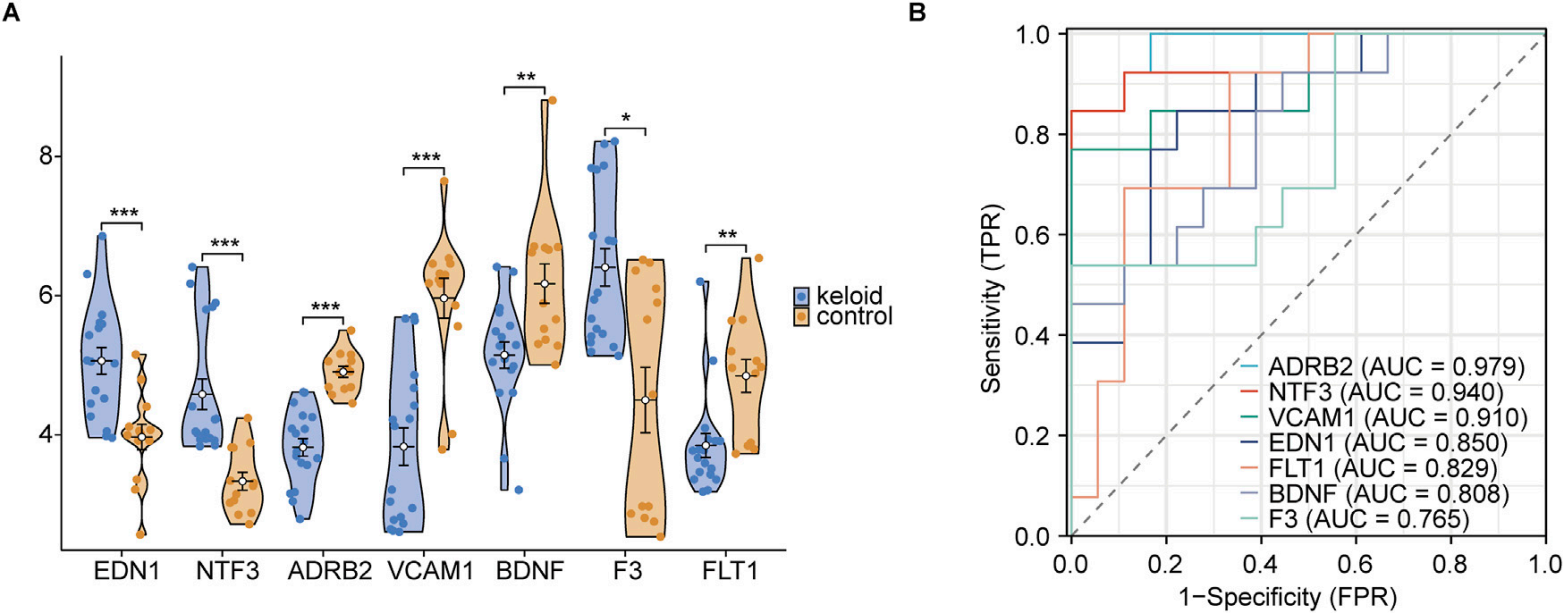
Group comparison and ROC diagnosis. **(A)** Expression levels of hub genes between keloid and normal groups. **(B)** ROC curve significance levels are indicated as follows: ns (not significant, *p* ≥ 0.05); * (*p* < 0.05, statistically significant); ** (*p* < 0.01, highly statistically significant); *** (*p* < 0.001, very highly statistically significant). The classification of the area under the curve (AUC) values is as follows: AUC values between 0.5 and 0.7 indicate low diagnostic accuracy, values between 0.7 and 0.9 indicate moderate accuracy, and values above 0.9 indicate high diagnostic accuracy.

In the violin plot (Figure 12A), EDN1, NTF3, ADRB2, and VCAM1 were found to be significantly upregulated in keloid tissue, with very high statistical significance (***, *p* < 0.001). Compared to the normal skin group, these genes showed markedly elevated expression in keloid tissue, suggesting their potential importance in the pathogenesis of keloids. Next, ROC curve analysis was used to rank the AUC values of the 7 hub genes. The highest AUC was observed for ADRB2 (AUC = 0.979), followed by NTF3 (AUC = 0.940), VCAM1 (AUC = 0.910), and EDN1 (AUC = 0.850). According to AUC classification criteria, ADRB2, NTF3, and VCAM1—with AUC values above 0.9—exhibited high diagnostic accuracy, while EDN1, with an AUC between 0.7 and 0.9, showed moderate diagnostic accuracy. Through these analyses, we further refined our focus to 4 key genes: ADRB2, NTF3, VCAM1, and EDN1.

### 3.6 Prediction of miRNA, transcription factor, and drug-gene interaction networks

**FIGURE 13:**
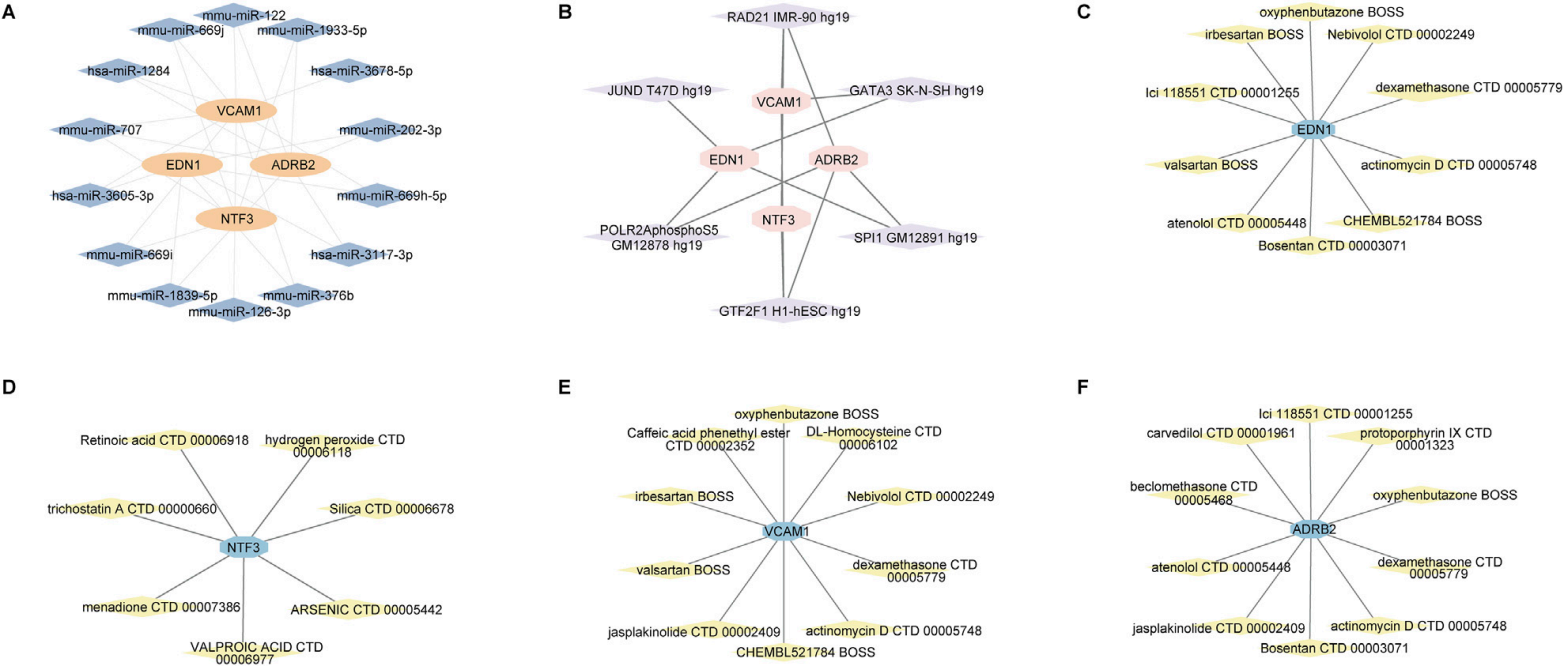
miRNA, transcription factor, and drug-gene interaction networks for the hub genes EDN1, NTF3, VCAM1 and ADRB2. **(A)** miRNA-gene interaction network. **(B)** Transcription factor-gene interaction network. **(C–F)** Drug-gene interaction networks.

Our analysis revealed the interaction networks associated with the hub genes EDN1, NTF3, ADRB2, and VCAM1, including miRNA, transcription factor, and drug-gene interactions. For example, miRNAs such as hsa-miR-335-5p and hsa-miR-30b-5p were found to be associated with multiple genes (Figure 13A). Key transcription factors, such as RAD21 and SP1, may regulate the expression of these hub genes (Figure 13B). In the drug-gene interaction networks, several compounds were predicted to interact with these hub genes. For instance, valproic acid is predicted to interact with NTF3, while dexamethasone is associated with VCAM1, suggesting that these genes could serve as potential therapeutic targets for modulating gene activity in the treatment of keloids (Figures 13C–F).

### 3.7 Immune cell infiltration and correlation analysis

**FIGURE 14:**
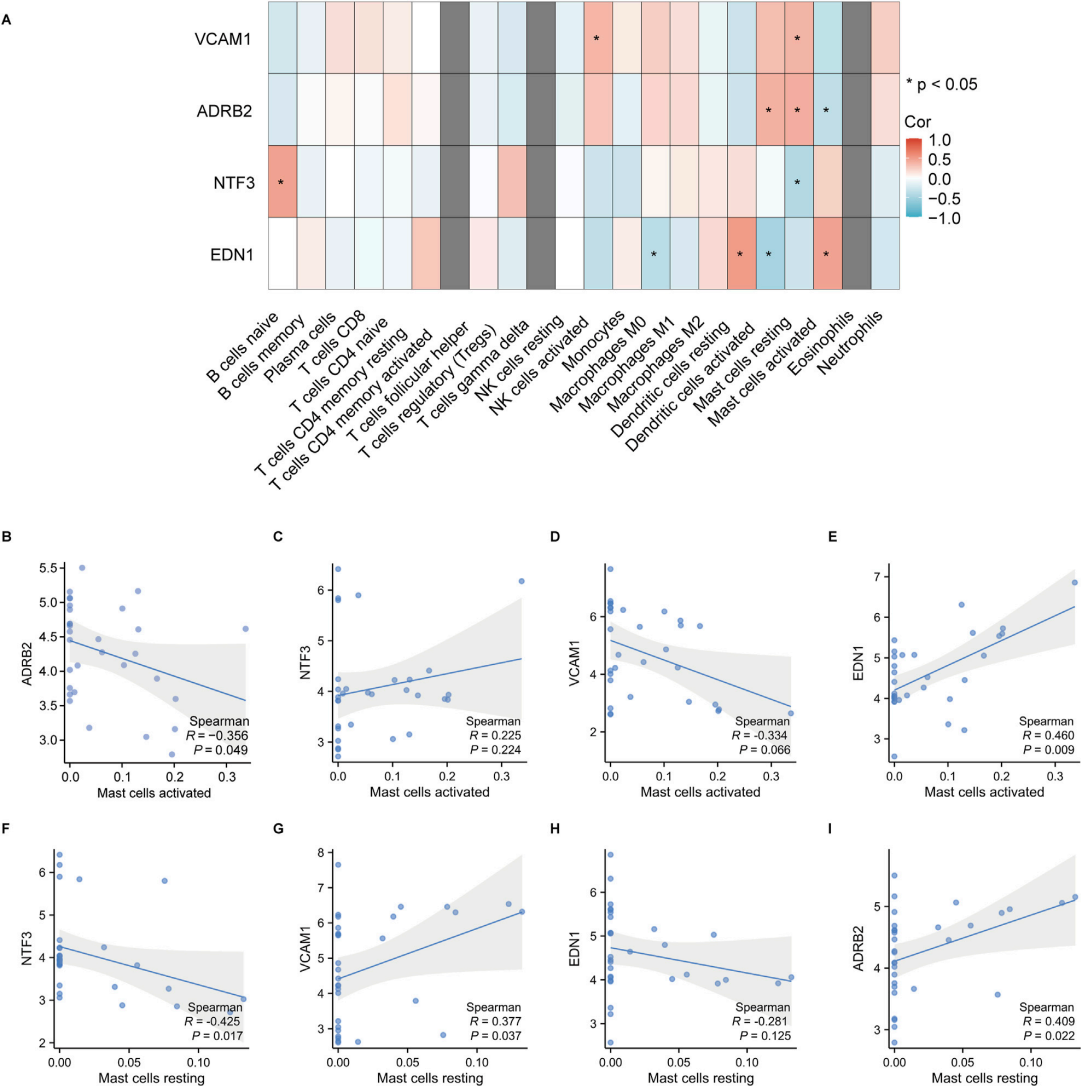
Correlation analysis between hub gene expression and immune cell infiltration in keloid tissues. **(A)** The heatmap shows the correlations between the hub genes VCAM1, ADRB2, NTF3 and EDN1 and various immune cell types, with significant correlations indicated by *. The correlation coefficients range from −1 to 1, with red representing positive correlations and blue representing negative correlations. **(B–I)** Correlation analysis between activated and resting mast cells and the hub genes, showing the Spearman correlation coefficients and p-values. Shaded areas represent the 95% confidence intervals.

The immune cell infiltration and correlation analysis in keloid tissues revealed significant associations between the expression of the hub genes ADRB2, NTF3, VCAM1, EDN1 and various immune cell types. The heatmap (Figure 14A) showed that among all immune cells, activated and resting mast cells exhibited the most significant correlations with these hub genes. Specifically, EDN1 had the strongest positive correlation with activated mast cells (Figure 14E, *r* = 0.460, *p* = 0.022), while NTF3 showed the strongest negative correlation with resting mast cells (Figure 14F, *r* = −0.425, *p* = 0.017). As a result, **EDN1** and **NTF3** were identified as the most important hub genes, warranting further investigation into their roles in the formation and progression of keloids.

#### 3.7.1 qPCR analysis of NTF3 and EDN1 in normal skin and keloid tissue

**FIGURE 15:**
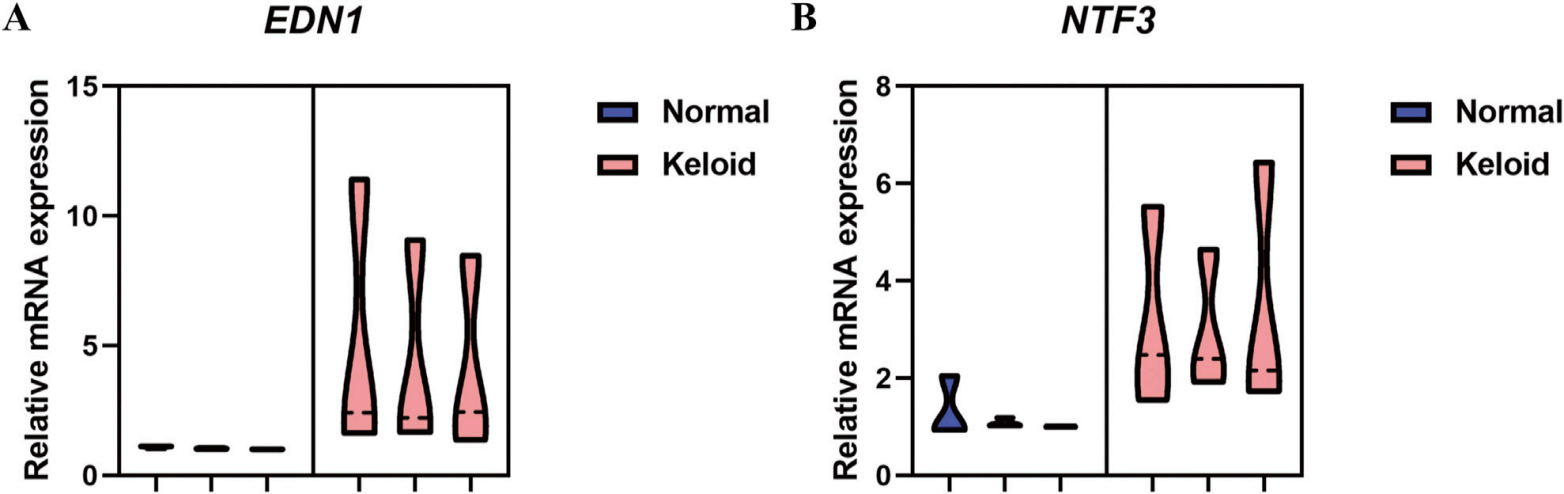
qPCR Analysis of Relative mRNA Expression Levels of NTF3 and EDN1 in Normal Skin and Keloid Tissue. **(A)** The violin plot represents the relative mRNA expression levels of EDN1 in normal skin (blue) and keloid tissue (pink). **(B)** The violin plot represents the relative mRNA expression levels of NTF3 in normal skin (blue) and keloid tissue (pink).

The qPCR analysis of NTF3 and EDN1 mRNA expression levels in normal skin and keloid tissue reveals a significant increase in both genes in keloid tissues. As illustrated in the violin plots (Figure 15), EDN1 expression is notably higher in keloid tissues compared to normal skin, with the keloid group showing a marked increase in mRNA levels. Similarly, NTF3 also exhibits elevated expression in keloid tissues, whereas its expression in normal skin remains minimal. These findings suggest that both EDN1 and NTF3 are significantly increased in keloid tissues, potentially implicating them in the pathological processes underlying keloid formation.

#### 3.7.2 Clinical and H&E-Stained comparison between normal skin and keloid tissue

**FIGURE 16:**
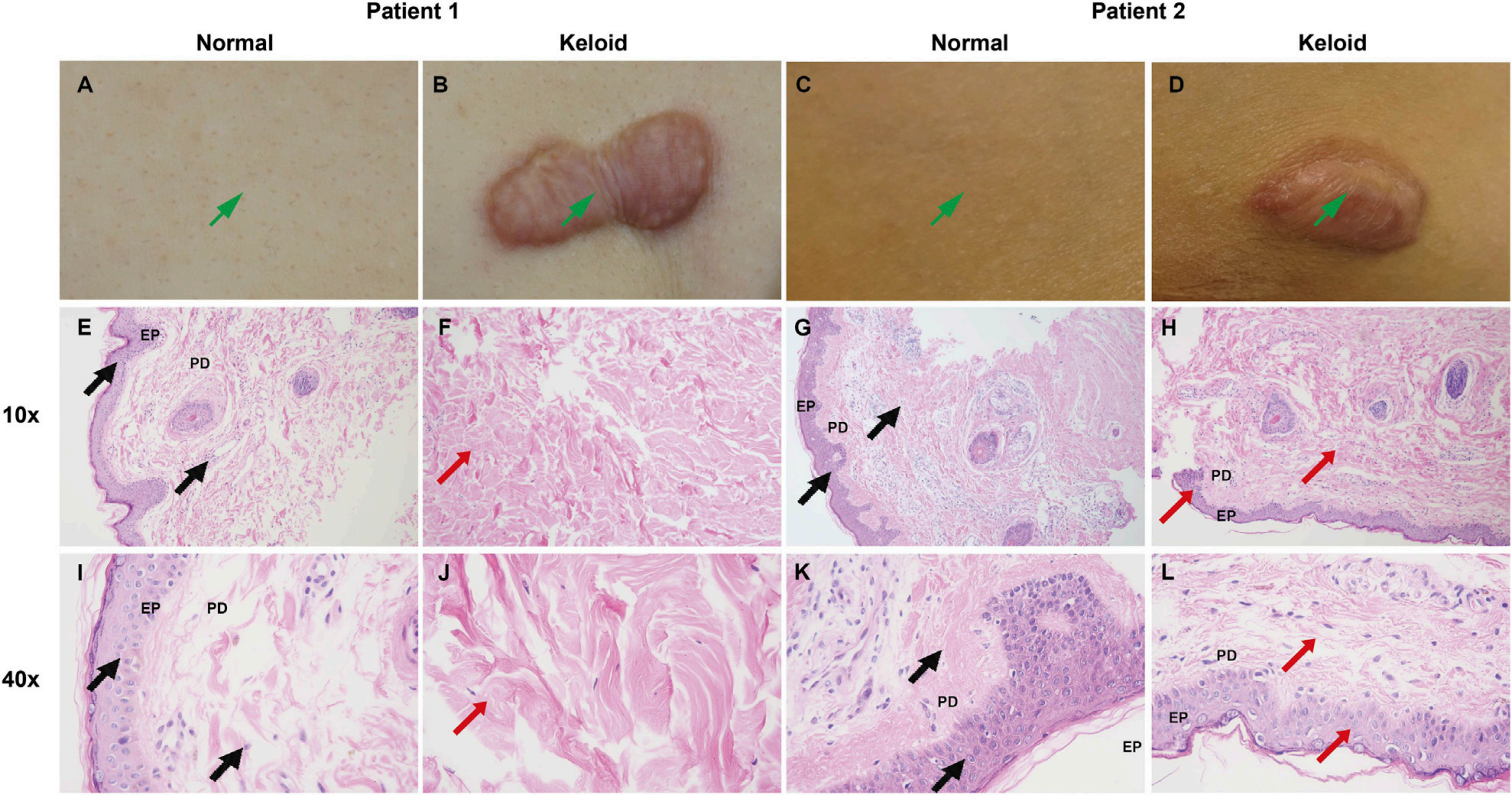
Clinical and H&E-stained comparison between normal skin and keloid tissue. **(A–D)** Clinical images of Patient 1 and Patient 2 showing normal skin **(A, C)** with a smooth surface, and keloid lesions **(B, D)** which appear raised and irregularly bordered. **(E–L)** H&E staining of normal skin **(E, G, I K)** and keloid tissue **(F, H, J, L)** at 10x and 40x magnification. Normal skin demonstrates well-organized collagen fibers (PD) and a uniform epidermal layer (EP) (**(E, G, I, K)**, marked by black arrows), while keloid tissue shows thickened, disorganized collagen bundles and irregular epidermal arrangement (**(F, H, J, L)**, marked by red arrows), consistent with fibrotic changes in keloids.

The clinical and histological comparison between normal skin and keloid tissue reveals marked differences in both structural and cellular organization. Clinically, as shown in the images of Patient 1 and Patient 2 (Figures 16A–D), keloid tissues appear as raised, firm, and irregularly bordered lesions (Figures 16B, D) compared to the smooth, even surface of normal skin (Figures 16A, C). Histologically, H&E-stained sections at 10x and 40x magnification demonstrate significant pathological alterations in keloid tissue. Normal skin shows well-organized collagen fibers (PD) and a uniform epidermis (EP) (Figures 16E, G, I, K), whereas keloid tissue exhibits thickened, densely packed, and disorganized collagen bundles (Figures 16F, H, J, L, indicated by red arrows), along with an irregular arrangement of epidermal cells, highlighting the characteristic fibrotic nature of keloids.

#### 3.7.3 IHC analysis of EDN1 and NTF3 expression in normal and keloid tissues

**FIGURE 17:**
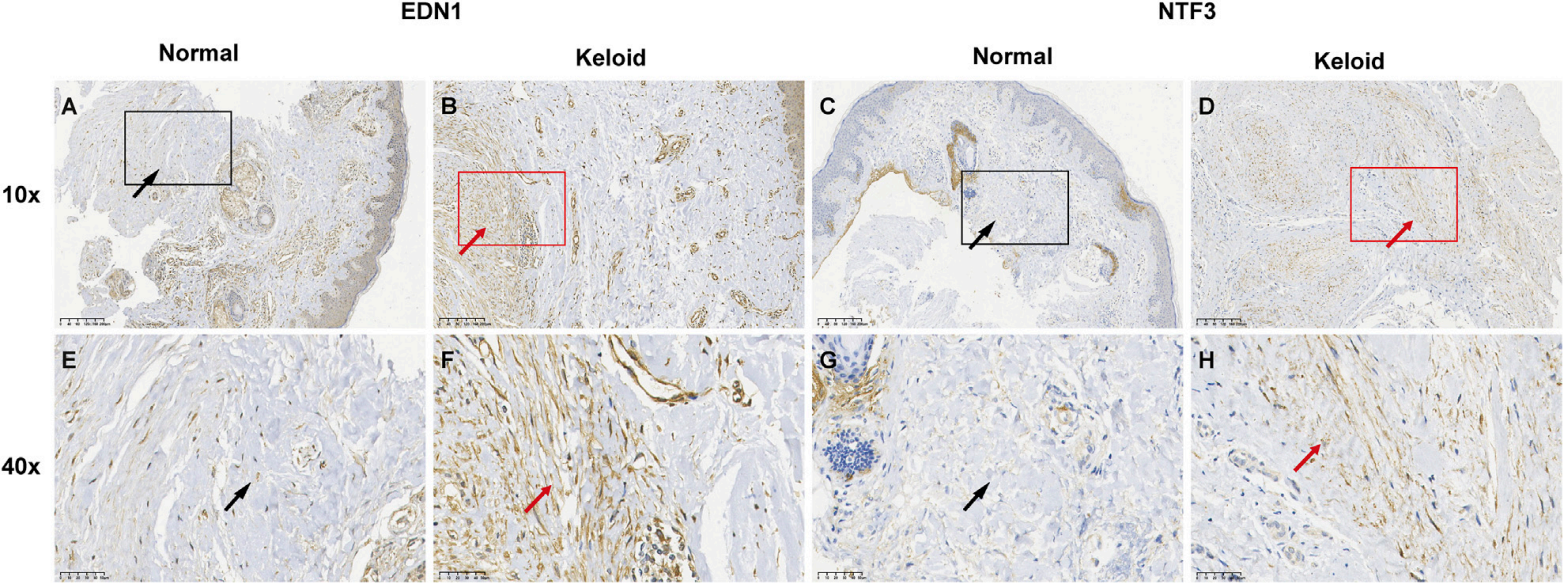
IHC analysis of EDN1 and NTF3 expression in normal skin and keloid tissues. **(A, C, E, G)** Normal skin tissue with moderate staining of EDN1 and NTF3, localized in the epidermis and adnexal structures, including hair follicles. **(B, D, F, H)** Keloid tissue showing significantly increased staining intensity of both EDN1 and NTF3. EDN1 displays strong staining in thickened collagen bundles and fibroblast-like cells, while NTF3 exhibits enhanced staining particularly in fibrotic areas of the keloid dermis. Red and black arrows indicate the regions of staining. Magnifications: 10x and 40x.

IHC analysis revealed distinct expression patterns of EDN1 and NTF3 in normal and keloid tissues. In normal skin, EDN1 showed moderate staining in the epidermis and around adnexal structures (Figures 17A, B), with clear localization in these regions. NTF3 expression was similarly localized with moderate staining in the epidermis and hair follicles (Figures 17C, D). In contrast, keloid tissues exhibited significantly higher expression levels of both EDN1 and NTF3. EDN1 showed intense staining throughout the thickened collagen bundles and fibroblast-like cells in the dermis (Figures 17E, F). Similarly, NTF3 exhibited stronger staining in the keloid dermis, particularly within the fibrotic regions (Figures 17G, H). These findings suggest that both EDN1 and NTF3 are upregulated in keloid tissues, potentially contributing to the pathological fibrosis and altered cellular environment characteristic of keloids.

## 4 Discussion

This study identified 13 oxidative stress-related differentially expressed genes (OSRDEGs) that were significantly expressed in keloid tissues through screening and multiple analyses. Among them, EDN1 and NTF3 were determined as key genes of interest. Their roles in oxidative stress processes in keloids were validated through WGCNA, functional enrichment analysis, qPCR, HE staining, IHC experiments, and external independent dataset validation.

### 4.1 Expression of EDN1 and NTF3 and their association with fibrosis

In keloid tissues, EDN1 and NTF3 were significantly upregulated, accompanied by prominent fibrotic features such as thickened and disorganized collagen fibers. Clinical photographs and HE staining results showed significant differences in fibrosis between keloid and normal skin tissues. Furthermore, IHC analysis confirmed the high expression of EDN1 and NTF3 in fibrotic regions, suggesting their critical roles in fibroblast activity and ECM remodeling.

### 4.2 Mechanistic roles of EDN1 and NTF3

EDN1 is a potent vasoconstrictive peptide secreted by vascular endothelial cells, primarily regulating vascular tone and blood pressure through ET-A and ET-B receptors (Banecki and Dora, 2023). Under pathological conditions, overexpression of EDN1 is associated with various cardiovascular diseases, such as hypertension and atherosclerosis (Sutton et al., 2019). EDN1 promotes the generation of reactive oxygen species (ROS) by activating NADPH oxidase, leading to increased oxidative stress, endothelial dysfunction, and vascular remodeling (Cai and Harrison, 2000). This process suggests that EDN1 plays a critical role in ROS generation and fibrosis. In keloids, EDN1 likely exacerbates inflammatory responses and fibroblast overproliferation by promoting ROS generation and activating the MAPK signaling pathway, resulting in abnormal ECM deposition (Dagamajalu et al., 2020).

NTF3 belongs to the neurotrophin family and primarily participates in the survival, differentiation, and regeneration of neurons (Lin et al., 2018). By binding to the TrkC receptor, NTF3 promotes neural system development and functional maintenance (Yang et al., 2020). Although most studies on NTF3 have focused on the nervous system, its role in immune regulation has attracted increasing attention. NTF3 has been shown to influence immune cell activation and proliferation by modulating the MAPK and PI3K/Akt signaling pathways (Yang et al., 2022). Current studies on NTF3 are predominantly limited to the nervous system, and its interaction with ROS remains underexplored, necessitating further investigation.

### 4.3 Fibrosis and inflammation interaction

This study found that the abnormal overexpression of key genes, such as EDN1 and NTF3, in keloid tissues is closely associated with significant fibrotic and inflammatory phenotypes. However, it remains controversial whether fibrosis and inflammation are the primary pathological drivers of keloid formation or secondary phenomena triggered by upstream events, such as genetic susceptibility, dysregulation of the TGF-β signaling pathway, or abnormal fibroblast proliferation (Ogawa and Regeneration, 2011). On the one hand, existing literature emphasizes that a positive feedback loop between persistent chronic inflammation and excessive extracellular matrix (ECM) deposition in keloids can sustain and even exacerbate excessive scar tissue overgrowth (McDougall et al., 2006). For instance, Ogawa et al. proposed that keloids can be considered the result of persistent chronic inflammation in the dermis, continuously stimulating fibroblast activation and inducing abnormal collagen deposition (Ogawa, 2017). On the other hand, some studies suggest that genetic factors or early gene mutations and signaling abnormalities are the initial pathogenic events, with fibrosis and inflammation potentially serving as amplifiers of these upstream pathological mechanisms, manifesting as secondary phenotypes at the tissue level (Zhang et al., 2009).

### 4.4 Association between oxidative stress and the MAPK signaling pathway

Functional enrichment analysis in this study revealed that key OSRDEGs (e.g., EDN1 and NTF3) were significantly enriched in signaling pathways closely associated with fibrosis and inflammation, including the MAPK signaling pathway, regulation of lymphocyte and monocyte proliferation, and cytokine activity regulation. ROS produced by OSRDEGs, as markers of oxidative stress, can activate key kinases in the MAPK pathway, such as ERK, JNK, and p38, through redox-sensitive signaling pathways (Son et al., 2011). These kinases further activate downstream transcription factors, such as AP-1 and NF-κB, which regulate gene expression to promote cell proliferation, inflammatory responses, and apoptosis (Geest and Coffer, 2009). In pathological conditions such as tumors and fibrosis, sustained activation of the MAPK pathway aggravates disease progression, induces persistent inflammatory responses, and promotes the increased secretion of cytokines such as TNF-α, IL-6, and IL-13 (Wagner and Nebreda, 2009). Persistently expressed inflammatory cytokines not only lead to overactivation of the immune system but also exacerbate tissue damage and fibrosis, such as abnormal fibroblast proliferation and excessive ECM deposition, which harden tissues and impair functionality (Kendall and Feghali-Bostwick, 2014).

### 4.5 Immune cell infiltration analysis

Immune cell infiltration analysis revealed significant correlations between activated and resting mast cells and several key genes, including EDN1, NTF3, ADRB2, and VCAM1. Activated mast cells regulate local inflammation and fibrosis by releasing inflammatory mediators such as histamine, tryptase, and various cytokines (Galli et al., 2008a). Mast cell activation can be triggered by the binding of IgE to its high-affinity receptor (FcεRI) or by ROS generated during oxidative stress, thereby exacerbating inflammatory responses (Gilfillan and Beaven, 2011). In damaged tissues, mast cells release inflammatory factors that attract other immune cells and promote fibrosis (Artuc et al., 2002). Resting mast cells are typically inactive and maintain tissue homeostasis, initiating inflammatory responses only upon receiving specific signals, such as allergens (Theoharides, 1990). In this study, EDN1 was positively correlated with activated mast cells, suggesting that EDN1 may promote mast cell activation, drive fibrosis progression in keloid tissues, and amplify inflammatory responses. Chemokines released by mast cells can further attract monocytes and lymphocytes, enhancing local inflammation (Galli et al., 2008b). Conversely, NTF3 was negatively correlated with resting mast cells, suggesting that NTF3 may inhibit their resting state, promote mast cell activation, and increase inflammatory responses. NTF3 may also regulate MAPK and PI3K/Akt signaling pathways, influencing immune cell activation and proliferation (Liu et al., 2021). In this study, the high expression of EDN1 and NTF3 was significantly correlated with fibrosis markers (COL1A1, TGFB1) and inflammatory factors (IL6, TNFA), supporting their dual regulatory roles in fibrosis and inflammation. This suggests that fibrosis and inflammation may form a mutually reinforcing relationship in keloids. On the one hand, persistent chronic inflammation stimulates excessive fibroblast proliferation and collagen deposition; on the other hand, excessive ECM deposition further recruits and activates immune cells, amplifying the inflammatory response (Nangole and Agak, 2019). However, to fully elucidate the sequence and interplay between fibrosis and inflammation in keloid progression, further longitudinal studies and *in vitro* and *in vivo* functional experiments are required. For instance, dynamic monitoring of these molecules and signaling pathways during the early stages of keloid development (e.g., early wound healing phases) could be insightful. Meanwhile, gene knockout, overexpression, and pharmacological intervention studies targeting specific genes could help clarify their precise roles in keloid initiation and progression. Our findings suggest that although fibrosis and inflammation may not be the initial triggers, they play indispensable roles in the development and maintenance of keloids. Thus, early therapeutic interventions targeting the inflammation-fibrosis pathway may offer new approaches for the clinical management and prevention of keloids.

### 4.6 Validation and WGCNA analysis

To enhance the representativeness and robustness of the study results, this research incorporated an independent external dataset, GSE7890, for validation. The results showed that although NTF3 was not significantly upregulated in the validation set, EDN1 and other genes (e.g., FLT1, FOXL2, VCAM1) displayed expression patterns consistent with the training set (GSE145725, GSE44270. The differences between the keloid and normal groups were statistically evaluated using the Mann-Whitney U test. Although some genes did not reach significance, likely due to the small sample size in the validation set, the overall results still support the importance of EDN1 and other genes in keloids. Furthermore, gene set enrichment analysis (GSEA) in both the training and validation datasets revealed significant enrichment of the IL-18 pathway, indicating the consistency of inflammatory signaling in keloids across different populations. WGCNA analysis identified EDN1 and NTF3 as key genes within the “brown” and “blue” modules, which were closely associated with fibrosis and inflammation. These modules were enriched with genes related to excessive ECM deposition and immune signaling. At the transcriptome-wide level, this further suggests that EDN1 and NTF3 may play dual regulatory roles in the fibrosis and inflammation processes of keloids. This analysis compensated for the limited statistical power of single-sample IHC studies and showed good concordance with immune cell-related heatmap findings. These findings further indicate that EDN1 and NTF3 may contribute to the development and progression of keloids by regulating fibrosis and inflammation.

### 4.7 Drug target prediction

The ROC analysis revealed that EDN1 and NTF3 exhibited high sensitivity and specificity in distinguishing keloid tissues from normal tissues, highlighting their potential value as biomarkers. This study also predicted miRNAs (e.g., hsa-miR-1264, mmu-miR-1933-5p), transcription factors (e.g., JUND, GATA3), and potential targeted drugs associated with EDN1 and NTF3. Previous studies have indicated that endothelin receptor antagonists, such as bosentan, can inhibit fibroblast activity and ECM deposition in various fibrotic diseases, warranting further evaluation in keloid models (Dhaun and Webb, 2019). Similarly, NTF3 may regulates MAPK/ERK and PI3K/Akt signaling pathways through TrkC receptors. Developing more specific TrkC inhibitors or antibodies, combined with localized drug delivery and multitarget approaches (e.g., p38/ERK inhibitors, anti-inflammatory agents), could potentially achieve better outcomes in suppressing the fibrosis-inflammation feedback loop (Kaplan and Miller, 2000).

### 4.8 Study limitations and future directions

Despite verifying the key roles of EDN1 and NTF3 through multiple analytical approaches, this study has some limitations. First, this study lacks functional experiments (e.g., gene knockdown or overexpression) to directly validate causal relationships. Second, the small sample size of the validation dataset might have affected the significance of some genes. Future studies will employ siRNA or CRISPR techniques in keloid-derived fibroblast or immune cell lines to observe their effects on ECM synthesis and inflammatory factor secretion. In addition, *in vivo* functional validation will be conducted as resources and conditions permit. Moreover, considering the ethnic and genetic susceptibilities of keloids, we provide demographic information (age, gender, lesion location, and ethnicity) of patients from the GSE145725 and GSE44270 datasets, presented in tabular form. Clinical keloid samples collected by our team were also summarized and presented in tabular format. Although these data do not fully encompass population variability, they enhance the extrapolative value and transparency of the study, laying the foundation for future multicenter or large-scale population studies.

## 5 Conclusion

This study integrated bioinformatics analysis and experimental validation to identify oxidative stress-related key genes associated with fibrosis and inflammatory responses in keloids. Using datasets from the GEO database (GSE145725, GSE44270, and GSE7890), differential expression analysis, WGCNA, GO/KEGG functional enrichment analysis, and GSEA were performed, leading to the identification of EDN1 and NTF3 as critical genes. These genes were found to be significantly dysregulated in keloid tissues and are potentially involved in oxidative stress, fibrosis, and inflammation. External dataset validation and WGCNA analysis further demonstrated the strong association of EDN1 and NTF3 with inflammation-related pathways, such as MAPK and IL-18 signaling. Additionally, EDN1 and NTF3 exhibited high correlations with fibrosis markers (COL1A1, TGFB1), inflammatory cytokines (IL6, TNFA), and immune cell infiltration (e.g., activated mast cells), suggesting their central regulatory roles in keloid-associated fibrosis and inflammation. Experimental validation using qPCR, HE staining, and IHC supported the bioinformatics findings. Although this study has not yet conducted gene knockdown or overexpression experiments to directly confirm causality, current evidence suggests that EDN1 and NTF3 have the potential to serve as diagnostic biomarkers and specific therapeutic targets. Future studies are needed to validate their molecular mechanisms and therapeutic interventions in larger, multicenter cohorts, as well as through *in vitro* and *in vivo* functional experiments. These efforts aim to provide novel insights and feasible strategies for the precise diagnosis and treatment of keloids.

## Data Availability

The data presented in the study are deposited in the GEO repository under accession numbers GSE145725, GSE44270, and GSE7890.

## References

[B1] Alder J. K. Guo N. Kembou F. Parry E. M. Anderson C. J. Gorgy A. I. (2011). Telomere length is a determinant of emphysema susceptibility. Am. J. Respir. Crit. Care Med. 184 (8), 904–912. 10.1164/rccm.201103-0520OC 21757622 PMC3208661

[B2] Allanore Y. Simms R. Distler O. Trojanowska M. Pope J. Denton C. P. (2015). Systemic sclerosis. Nat. Rev. Dis. Prim. 1 (1), 15002–15021. 10.1038/nrdp.2015.2 27189141

[B3] Antar S. A. Ashour N. A. Marawan M. E. Al-Karmalawy A. (2023). Fibrosis: types, effects, markers, mechanisms for disease progression, and its relation with oxidative stress, immunity, and inflammation. Int. J. Mol. Sci. 24 (4), 4004. 10.3390/ijms24044004 36835428 PMC9963026

[B4] Armanios M. Blackburn E. H. (2012). The telomere syndromes. Nat. Rev. Genet. 13 (10), 693–704. 10.1038/nrg3246 22965356 PMC3548426

[B5] Artuc M. Steckelings U. M. Henz B. M. (2002). Mast cell–fibroblast interactions: human mast cells as source and inducers of fibroblast and epithelial growth factors. J. Invest. Dermatol. 118 (3), 391–395. 10.1046/j.0022-202x.2001.01705.x 11874475

[B6] Banecki K. M. R. M. Dora K. A. (2023). Endothelin-1 in health and disease. Int. J. Mol. Sci. 24 (14), 11295. 10.3390/ijms241411295 37511055 PMC10379484

[B7] Cai H. Harrison D. G. (2000). Endothelial dysfunction in cardiovascular diseases: the role of oxidant stress. Circ. Res. 87 (10), 840–844. 10.1161/01.res.87.10.840 11073878

[B8] Dagamajalu S. Rex D. A. B. Gopalakrishnan L. Karthikkeyan G. Gurtoo S. Modi P. K. (2020). A network map of endothelin mediated signaling pathway. J. Cell Commun. Signal. 15 (2), 277–282. 10.1007/s12079-020-00581-4 32915369 PMC7990991

[B9] Darby I. A. Laverdet B. Bonté F. Desmoulière A. (2014). Fibroblasts and myofibroblasts in wound healing. Clin. Cosmet. Investig. Dermatol 7 301–311. 10.2147/CCID.S50046 PMC4226391 25395868

[B10] Dhaun N. Webb D. J. (2019). Endothelins in cardiovascular biology and therapeutics. Nat. Rev. Cardiol. 16 (8), 491–502. 10.1038/s41569-019-0176-3 30867577

[B11] Forman H. J. Zhang H. Rinna A. (2009). Glutathione: overview of its protective roles, measurement, and biosynthesis. Mol. Asp. Med. 30 (1-2), 1–12. 10.1016/j.mam.2008.08.006 PMC2696075 18796312

[B12] Galli S. J. Grimbaldeston M. Tsai M. (2008a). Immunomodulatory mast cells: negative, as well as positive, regulators of immunity. Nat. Rev. Immunol. 8 (6), 478–486. 10.1038/nri2327 18483499 PMC2855166

[B13] Galli S. J. Tsai M. Piliponsky A. M. (2008b). The development of allergic inflammation. Nature 454 (7203), 445–454. 10.1038/nature07204 18650915 PMC3573758

[B14] Geest C. R. Coffer P. J. (2009). MAPK signaling pathways in the regulation of hematopoiesis. J. Leukoc. Biol. 86 (2), 237–250. 10.1189/jlb.0209097 19498045

[B15] Gilfillan A. M. Beaven M. A. (2011). Regulation of mast cell responses in health and disease. Crit. Rev. Immunol. 31 (6), 475–529. 10.1615/critrevimmunol.v31.i6.30 22321108 PMC3395887

[B16] Hecker L. Logsdon N. J. Kurundkar D. Kurundkar A. Bernard K. Hock T. (2014). Reversal of persistent fibrosis in aging by targeting Nox4-Nrf2 redox imbalance. Sci. Transl. Med. 6 (231), 231–247. 10.1126/scitranslmed.3008182 PMC4545252 24718857

[B17] Hecker L. Thannickal V. J. (2011). Nonresolving fibrotic disorders: idiopathic pulmonary fibrosis as a paradigm of impaired tissue regeneration. Am. J. Med. Sci. 341 (6), 431–434. 10.1097/MAJ.0b013e31821a9d66 21613929

[B18] Hinz B. (2007). Formation and function of the myofibroblast during tissue repair. J. Invest. Dermatol. 127 (3), 526–537. 10.1038/sj.jid.5700613 17299435

[B19] Jiang D. Rinkevich Y. (2020). Scars or regeneration? dermal fibroblasts as drivers of diverse skin wound responses. Int. J. Mol. Sci. 21 (2), 617. 10.3390/ijms21020617 31963533 PMC7014275

[B20] Kaplan D. R. Miller F. D. (2000). Neurotrophin signal transduction in the nervous system. Curr. Opin. Neurobiol. 10 (3), 381–391. 10.1016/s0959-4388(00)00092-1 10851172

[B21] Kendall R. T. Feghali-Bostwick C. A. (2014). Fibroblasts in fibrosis: novel roles and mediators. Front. Pharmacol. 5, 123. 10.3389/fphar.2014.00123 24904424 PMC4034148

[B22] Kim E. K. Choi E.-J. (2010). Pathological roles of MAPK signaling pathways in human diseases. Biochim. Biophys. Acta 1802 (4), 396–405. 10.1016/j.bbadis.2009.12.009 20079433

[B23] Kuilman T. Michaloglou C. Mooi W. J. Peeper D. S. (2010). The essence of senescence. Genes Dev. 24 (22), 2463–2479. 10.1101/gad.1971610 21078816 PMC2975923

[B24] Lin Y.-M. J. Hsin I.-L. Sun H. S. Lin S. Lai Y.-L. Chen H.-Y. (2018). NTF3 is a novel target gene of the transcription factor POU3F2 and is required for neuronal differentiation. Mol. Neurobiol. 55, 8403–8413. 10.1007/s12035-018-0995-y 29549646 PMC6153716

[B25] Liu B. Zhang Y. Yang Z. Liu M. Zhang C. Zhao Y. (2021). ω-3 DPA protected neurons from neuroinflammation by balancing microglia M1/M2 polarizations through inhibiting NF-κB/MAPK p38 signaling and activating neuron-BDNF-PI3K/AKT pathways. Mar. Drugs 19 (11), 587. 10.3390/md19110587 34822458 PMC8619469

[B26] McDougall S. Dallon J. Sherratt J. Maini P. (2006). Fibroblast migration and collagen deposition during dermal wound healing: mathematical modelling and clinical implications. Philos. Trans. A Math. Phys. Eng. Sci. 364 (1843), 1385–1405. 10.1098/rsta.2006.1773 16766351

[B27] Miyazono K. (2000). TGF-beta signaling by Smad proteins. Cytokine Growth Factor Rev. 11 (1), 15–22. 10.1016/S1359-6101(99)00025-8 10708949

[B28] Morgan M. J. Liu Z. (2011). Crosstalk of reactive oxygen species and NF-κB signaling. Cell Res. 21 (1), 103–115. 10.1038/cr.2010.178 21187859 PMC3193400

[B29] Nakashima M. Chung S. Takahashi A. Kamatani N. Kawaguchi T. Tsunoda T. (2010). A genome-wide association study identifies four susceptibility loci for keloid in the Japanese population. Nat. Genet. 42 (9), 768–771. 10.1038/ng.645 20711176

[B30] Nangole F. W. Agak G. W. (2019). Keloid pathophysiology: fibroblast or inflammatory disorders? JPRAS Open 22, 44–54. 10.1016/j.jpra.2019.09.004 32051841 PMC7015170

[B31] Ogawa R. (2011). Mechanobiology of scarring. Wound Repair Regen. 19, s2–s9. 10.1111/j.1524-475X.2011.00707.x 21793962

[B32] Ogawa R. (2017). Keloid and hypertrophic scars are the result of chronic inflammation in the reticular dermis. Int. J. Mol. Sci. 18 (3), 606. 10.3390/ijms18030606 28287424 PMC5372622

[B33] Schäfer M. Werner S. (2008). Oxidative stress in normal and impaired wound repair. Pharmacol. Res. 58 (2), 165–171. 10.1016/j.phrs.2008.06.004 18617006

[B34] Shih B. Garside E. McGrouther D. A. Bayat A. (2010). Molecular dissection of abnormal wound healing processes resulting in keloid disease. Wound Repair Regen. 18 (2), 139–153. 10.1111/j.1524-475X.2009.00553.x 20002895

[B35] Son Y. Cheong Y.-K. Kim N.-H. Chung H.-T. Kang D. G. Pae H.-O. (2011). Mitogen-activated protein kinases and reactive oxygen species: how can ROS activate MAPK pathways? J. Signal Transduct. 2011 (1), 792639. 10.1155/2011/792639 21637379 PMC3100083

[B36] Sundaresan N. R. Vasudevan P. Zhong L. Kim G. Samant S. Parekh V. (2012). The sirtuin SIRT6 blocks IGF-Akt signaling and development of cardiac hypertrophy by targeting c-Jun. Nat. Med. 18 (11), 1643–1650. 10.1038/nm.2961 23086477 PMC4401084

[B37] Sutton G. Pugh D. Dhaun N. (2019). Developments in the role of endothelin-1 in atherosclerosis: a potential therapeutic target? Am. J. Hypertens. 32 (9), 813–815. 10.1093/ajh/hpz091 31145445 PMC6694011

[B38] Theoharides T. (1990). Mast cells: the immune gate to the brain. Life Sci. 46 (9), 607–617. 10.1016/0024-3205(90)90129-f 2407920

[B39] Wagner E. F. Nebreda Á. R. (2009). Signal integration by JNK and p38 MAPK pathways in cancer development. Nat. Rev. Cancer 9 (8), 537–549. 10.1038/nrc2694 19629069

[B40] Yang Q.-X. Liu T. Yang J.-L. Liu F. Chang L. Che G.-L. (2020). Low expression of NTF3 is associated with unfavorable prognosis in hepatocellular carcinoma. Int. J. Clin. Exp. Pathol. 13 (9), 2280–2288. 33042332 PMC7539881

[B41] Yang Z. Zhang H. Yin M. Cheng Z. Jiang P. Feng M. (2022). Neurotrophin3 promotes hepatocellular carcinoma apoptosis through the JNK and P38 MAPK pathways. Int. J. Biol. Sci. 18 (15), 5963–5977. 10.7150/ijbs.72982 36263167 PMC9576519

[B42] Zhang Q. Yamaza T. Kelly A. P. Shi S. Wang S. Brown J. (2009). Tumor-like stem cells derived from human keloid are governed by the inflammatory niche driven by IL-17/IL-6 axis. PLoS One 4 (11), e7798. 10.1371/journal.pone.0007798 19907660 PMC2771422

