# Supplementary material for: EDN1 and NTF3 in keloid pathogenesis: computational and experimental evidence as novel diagnostic biomarkers for fibrosis and inflammation
Source: Front Genet. 2025 Feb 20;16:1516451. doi: 10.3389/fgene.2025.1516451 (PMC11882859; doi:10.3389/fgene.2025.1516451)
Supplement: Supplementary file 2 [file Table1.docx]

Table 1. Primers used

| Gene | Forward | Reverse |
| --- | --- | --- |
| *GAPDH* | GTGGAGTCCACTGGCGTCTTC | CTGATGATCTTGAGGCTGTTGTCA |
| *EDN1* | TGCCTTTTCTCCCCGTTAAA | CGCCTAAGACTGCTGTTTCT |
| *NTF3* | AGAGTTGAAGCTCCTCTCCC | CTTGTTCACCTGTAAGATCGTGG |
