# Supplementary figures and images for: EDN1 and NTF3 in keloid pathogenesis: computational and experimental evidence as novel diagnostic biomarkers for fibrosis and inflammation
Source: Front Genet. 2025 Feb 20;16:1516451. doi: 10.3389/fgene.2025.1516451 (PMC11882859; doi:10.3389/fgene.2025.1516451)

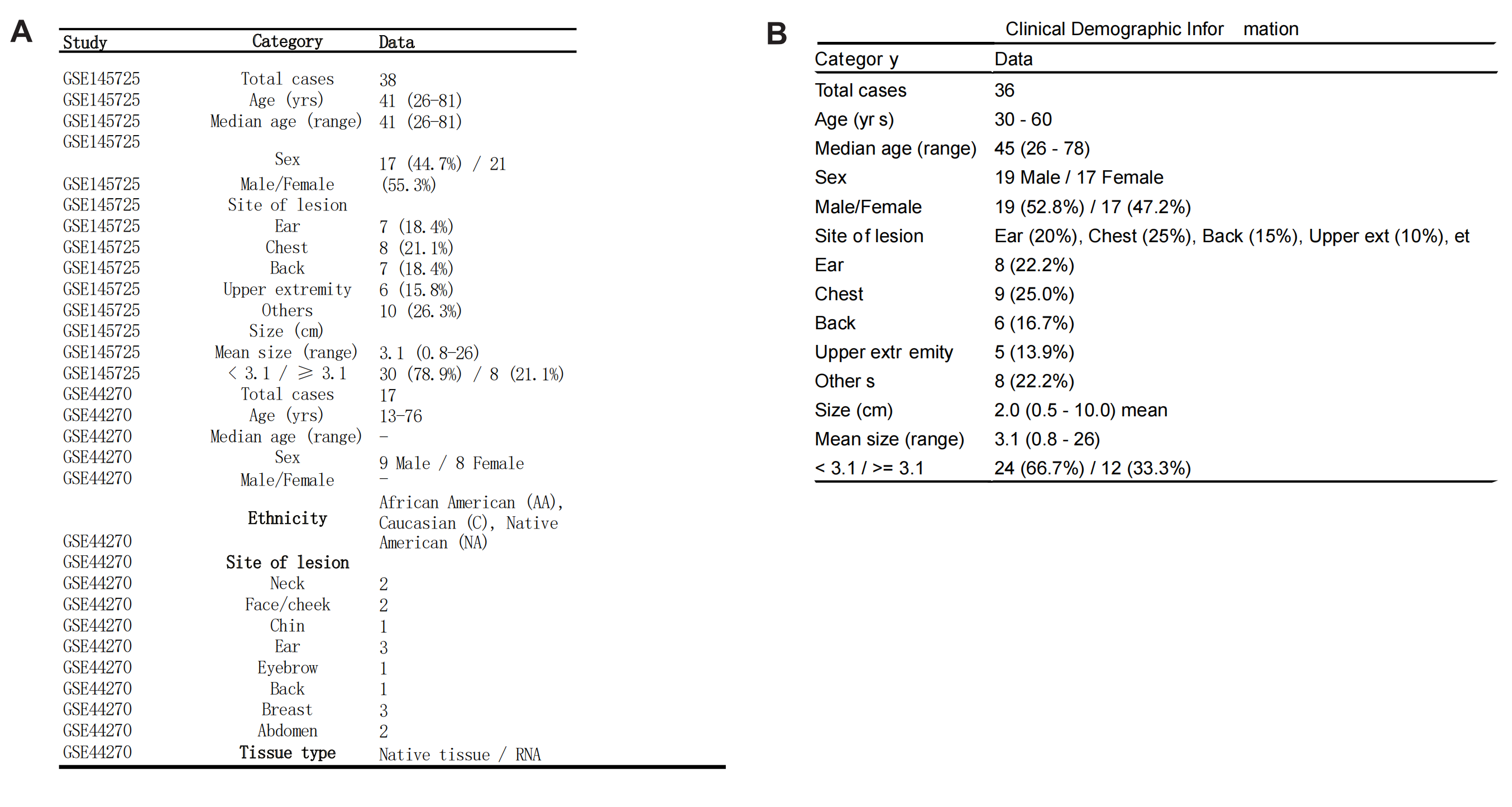

Supplement: Supplementary file 3 [file Image3.tif]

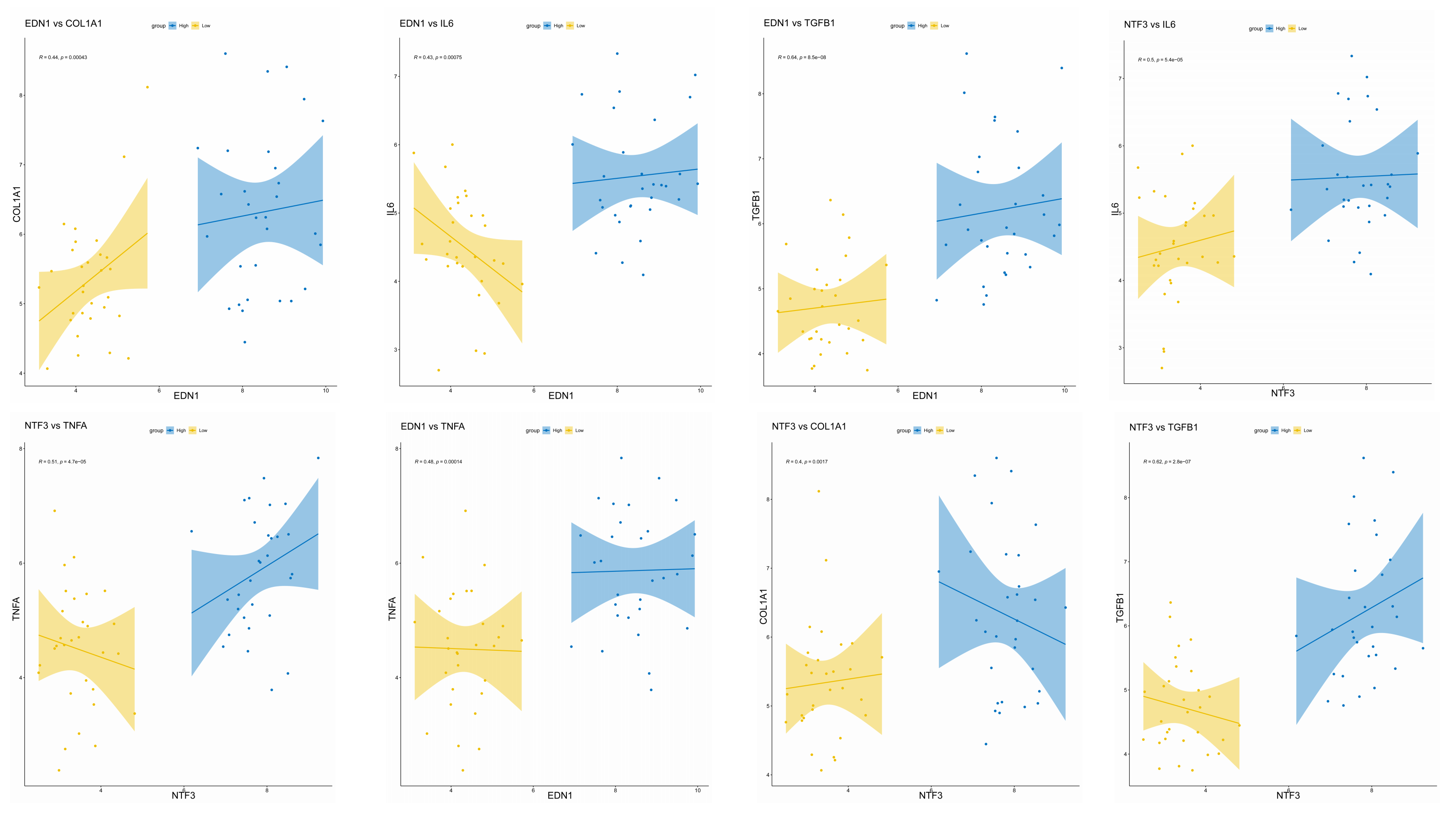

Supplement: Supplementary file 4 [file Image2.tif]

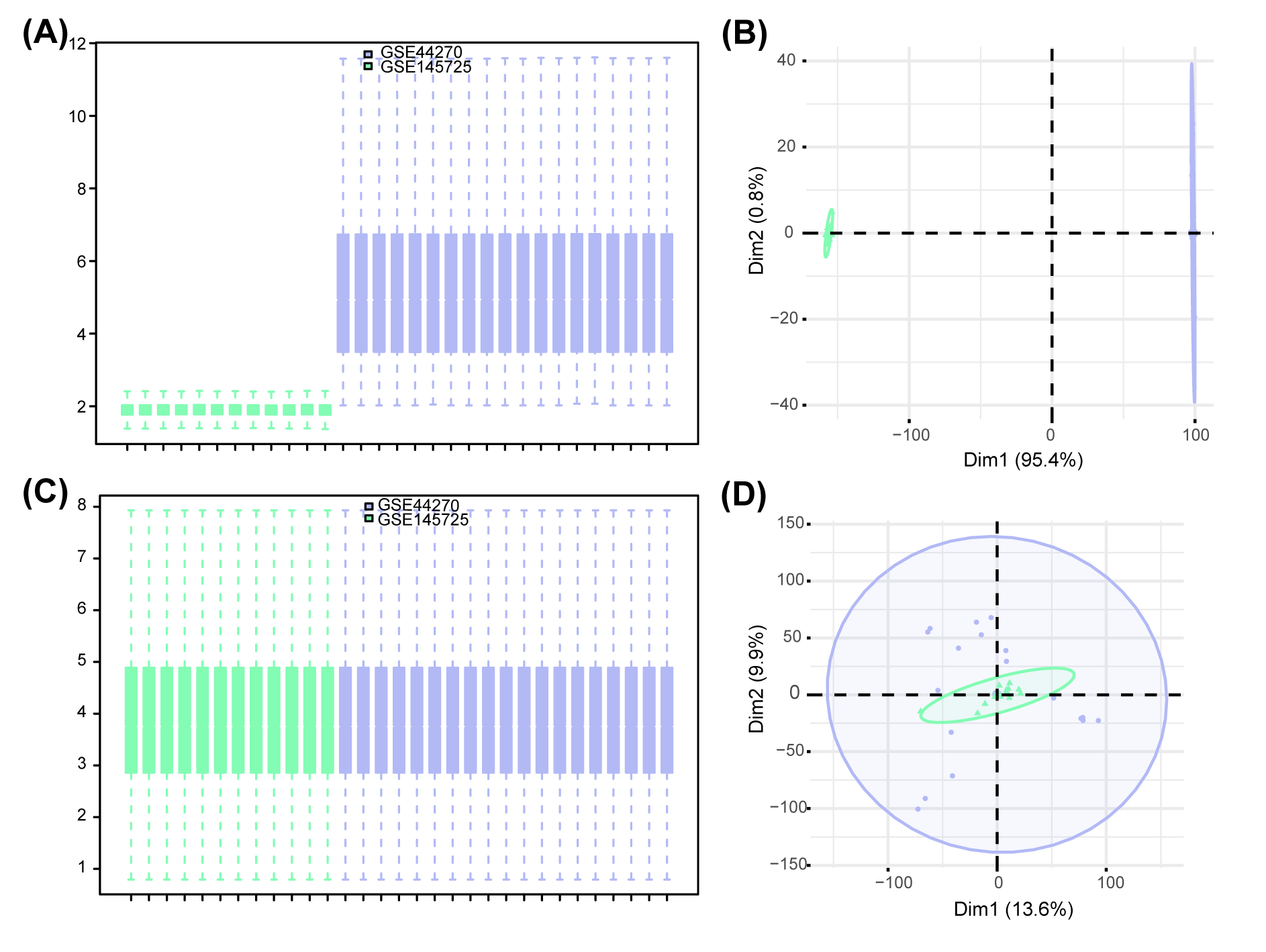

Supplement: Supplementary file 5 [file Image1.tif]
